# Phospholipid Scramblase 1 (PLSCR1) Regulates Interferon-Lambda Receptor 1 (IFN-λR1) and IFN-λ Signaling in Influenza A Virus (IAV) Infection

**DOI:** 10.1101/2024.11.20.624469

**Published:** 2024-11-21

**Authors:** Alina X. Yang, Lisa Ramos-Rodriguez, Parand Sorkhdini, Dongqin Yang, Carmelissa Norbrun, Sonoor Majid, Yong Zhang, Michael J. Holtzman, David F. Boyd, Yang Zhou

**Affiliations:** 1 Department of Molecular Microbiology and Immunology, Brown University, RI, USA; 2 Division of Pulmonary and Critical Care Medicine, Washington University School of Medicine in St. Louis, MO, USA; 3 Department of Molecular, Cell and Developmental Biology, University of California Santa Cruz, CA, USA

## Abstract

Phospholipid scramblase 1 (PLSCR1) is an antiviral interferon-stimulated gene (ISG) that has several known anti-influenza functions such as interfering with viral nuclear import, regulating toll-like receptor (TLR) 9 and potentiating the expression of other ISGs. However, the exact mechanisms of anti-flu activity of PLSCR1 in relation to its expression compartment and enzymatic activity, and the molecular and cellular mechanisms involved have not been completely explored. Moreover, only limited animal models have been studied to delineate its role at the tissue level in influenza infections. We hypothesize that PLSCR1 protects hosts against IAV infection by regulating type 3 interferon (IFN-λ) signaling pathways. Our results showed that Plscr1 expression was highly induced by IAV infection *in vivo* and in epithelial cells treated with IFN-λ. We found that *Plscr1* knockout (KO) mice exhibited exacerbated body weight loss, decreased survival rates, heightened viral replication, and increased lung damage. Interestingly, transcriptomic analyses demonstrated that Plscr1 was required for type 3 interferon receptor (Ifn-λr1) expression, and impaired expression of Ifn-λr1 and downstream ISGs may be responsible for delayed viral clearance in Plscr1 KO mice. In addition, Plscr1 interacted with Ifn-λr1 within the epithelial compartment following IAV infection, suggesting Plscr1 may modulate IFN-λ signaling via protein-protein interactions. Finally, single-cell RNA sequencing data indicated that *Plscr1* expression was significantly upregulated in ciliated airway epithelial cells in mice following IAV infection. Consistently, *Plscr1*^*floxStop*^*Foxj1-Cre*^*+*^ mice with ciliated epithelial cell-specific Plscr1 overexpression showed reduced susceptibility, less inflammation and enhanced Ifn-λr1 expression in IAV infection. Our research will elucidate virus-host interactions and pave the way for the development of novel anti-influenza drugs that target human elements like PLSCR1, thereby mitigating the emergence of drug-resistant IAV strains.

## INTRODUCTION

Influenza A virus (IAV) is highly contagious and causes acute respiratory infectious disease transmitted by virus-containing droplets. Its segmented gene and wide host range facilitate frequent antigenic shift during coinfection, posing a significant threat to human health. IAV has caused several pandemics in human history, and seasonal flu remains a major health burden in present days, despite annual vaccination efforts[1]. Existing anti-flu drugs mainly target influenza virus proteins: 1) penetration and shelling inhibitors, such as Gocovri (amantadine) and Flumadine (Rimantadine); 2) neuraminidase inhibitors, such as Relenza (zanamivir), Tamiflu (oseltamivir phosphate), Rapivab (peramivir) and Xofluza (baloxavir marboxil)[2]. However, the emergence of drug-resistant variant strains is common. Moreover, these direct antivirals have a short therapeutic window and are the most effective only if given within the first 48 hours after the initial infection[2]. In addition, IAV-induced acute inflammation could persist, leading to severe complications such as life-threatening pneumonia, immunopathology, and acute respiratory distress syndrome (ARDS). Therefore, there is a pressing need to understand host immune responses and develop anti-influenza drugs targeting host factors.

At the center of anti-flu immunity are the interferon pathways. Discovered a mere two decades ago, type 3 interferons were initially perceived as redundant to type 1 interferons, given their shared intracellular signaling pathways and antiviral activities. However, recent studies have revealed their unique properties, notably their signaling through a pair of heterodimer receptors (IFN-λR1/IL-10R2) distinct from type 1 interferon receptor complex (IFN-αR1/IFN-αR2)[3, 4]. First of all, IFN-λ exhibits a constrained expression pattern compared to type 1 interferons. While type 1 interferons are expressed almost by all cells, IFN-λ is primarily secreted by cells at barrier surfaces, such as respiratory and gastrointestinal epithelial cells, dendritic cells and macrophages[5, 6]. Moreover, their receptor distributions are vastly different. IFN-αR1/IFN-αR2 complex is present on nearly all cell types, but IFN-λR1/IL-10R2 complex is expressed exclusively on epithelial cells and few immune cells, including neutrophils and subsets of dendritic cells[7, 8]. Most importantly, IFN-λ is produced earlier than type 1 interferons in IAV infection and can elicit effective antiviral responses without inflammation such as the release of tissue-damaging mediators such as tumor necrosis factor (TNF) and IL-1β from neutrophils. In fact, only under a high dose of IAV are type 1 interferons detected, contributing to tissue immunopathology [9]. Another exclusive anti-flu mechanism of IFN-λ is its role in preventing viral transmission from the upper airways to the lungs[10]. Taken together, IFN-λ provides a non-redundant front-line shield against influenza virus.

PLSCR1 is the most studied member of the phospholipid scramblase protein family. It is a type II transmembrane protein generally located on the cell membrane, but can be imported into the nucleus and act as a transcriptional factor to regulate several gene expressions by directly binding to their promoter regions[11, 12]. PLSCR1 is detected in all tissues and has a relatively high expression in lung. Although it is universally expressed across various cell types in the lung including alveolar epithelial cells and lymphocytes, macrophages have the highest expression, followed by endothelial cells and respiratory ciliated cells[13]. The main function of PLSCR1 is to catalyze Ca2+ dependent, ATP independent, bidirectional and non-specific translocation of phospholipids between inner & outer leaflet of plasma membrane. Scrambling of membrane phospholipids results in the externalization of phosphatidylserine (PS), which acts as a docking site for many biological processes including coagulation, apoptosis, and activation[14]. Intriguingly, PLSCR1 is also an antiviral ISG, as its expression can be highly induced by type 1 and 2 interferons in various viral infections[15, 16]. However, whether its expression can be similarly induced by type 3 interferon has not been studies yet.

Previous studies have described some critical anti-influenza activities of PLSCR1. For example, PLSCR1 interacts with the nucleoprotein (NP) of IAV, impairing its nuclear import and thereby suppressing virus replication in A549 cells[17]. In addition, Plscr1 competes with immunoglobulin-like domain-containing receptor 1 (ILDR1) for NP binding, inhibiting swine influenza virus (SIV) infection in mice[18]. Besides direct interactions with the virus, PLSCR1 interacts with TLR9 and regulates its trafficking and ability to induce type I interferon production in plasmacytoid dendritic cells[19]. Furthermore, PLSCR1 is required to potentiate the expression of numerous other ISGs in response to IFN-β in Hey1B cells, including p56, ISG15 and OAS[20]. The goals of this project are to determine the roles of Plscr1 in an IAV-infected mouse model, to implicate its involvement in IFN-λ signaling, and to elucidate the cell types responsible for Plscr1-mediated anti-influenza activities.

To address this knowledge gap, we have employed a systematic approach, leveraging both global and cell type-specific *Plscr1*^−/−^ and *Plscr1* knock-in overexpression mice infected with mouse-adapted human IAV. Human respiratory epithelial cell lines harboring PLSCR1 mutations have also been used to elucidate the subcellular roles of PLSCR1. Our findings demonstrate that Plscr1 is an IFN-λ-stimulated gene, and it protects mice against IAV Infection by regulating *IFN-λR1* gene expression in the nucleus and interacting with IFN-λR1 protein on the cell membrane. Our research will aid in the understanding of virus-host interactions and the development of novel anti-influenza therapeutics targeting human components, preventing the emergence of drug-resistant IAV strains.

## RESULTS

### Plscr1 Suppresses Viral Replication and Protects Mice in IAV Infection

**Figure 1.**
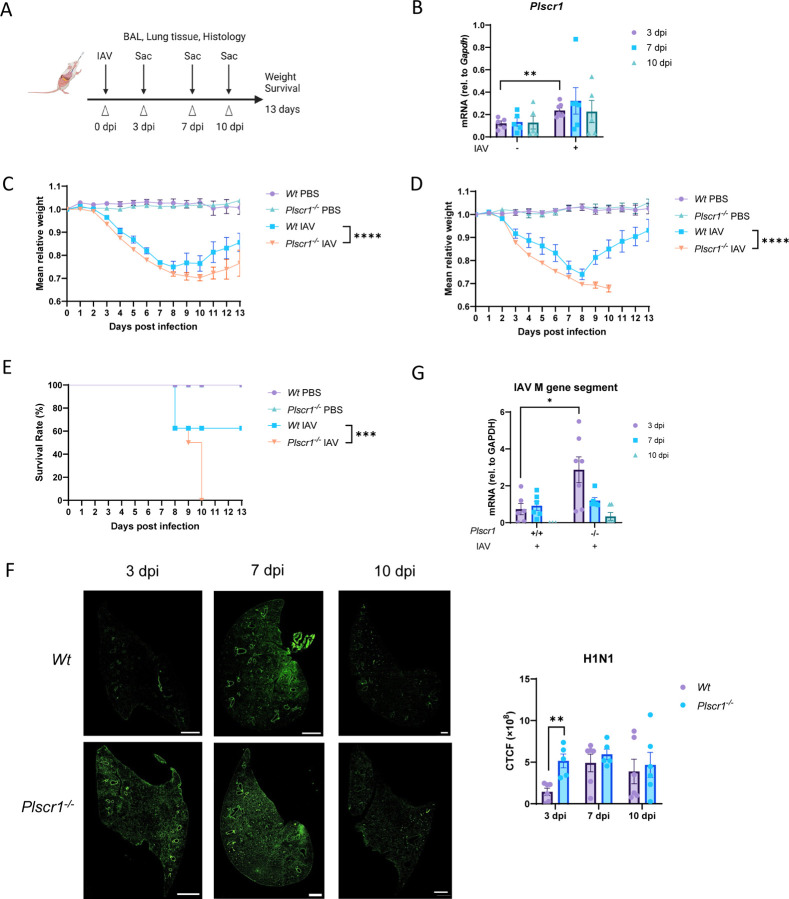
Increased Susceptibility of Plscr1−/− Mice to Influenza Virus Infection *Wt* and *Plscr1*^−/−^ mice were exposed to sublethal (300 pfu, B, C, G and F) or lethal (900 pfu, D and E) IAV (WSN) infection. (A) Scheme of experiment. (B) Whole lungs were analyzed for *Plscr1* RNA by qRT-PCR. (C and D) Mean relative weight of mice post sublethal or lethal infection. (E) Survival rate of mice post lethal IAV infection. (F) Representative staining for H1N1 in lungs. The scale bars represent 1 mm. Quantification was performed using ImageJ. (G) Viral RNA load in the lungs was assessed by quantifying M gene by qRT-PCR. Data are expressed as mean ± SEM of n = 30 mice/group for weight loss post sublethal infection and n=8 mice/group for weight loss and survival rate post lethal infection. For the rest analysis, n= 5–10 mice/group. All data were pooled from three independent experiments. *p < 0.05, **p < 0.01, ***p<0.001, ****p<0.0001. dpi, days post infection.

To uncover any anti-influenza functions of Plscr1 in mice, we employed *Wt* and *Plscr1*^−/−^ mice and exposed them to IAV infection. We pursued a systematic approach including multiple endpoints and parameters to monitor mouse health, viral replication and dissemination, antiviral and inflammatory responses, and tissue damage (Figure 1A). We established 300 plaque-forming unit (pfu) of IAV as the sublethal dose, and 900 pfu as the lethal dose resulting in ~50% mortality in *Wt* mice (LD50). Significantly higher *Plscr1* expression was observed in *Wt* infected mice at 3 days post infection (dpi) (Figure 1B), suggesting that Plscr1 is induced and potentially functions as an antiviral ISG in IAV infection *in vivo*.

Weight loss generally started at 3 dpi for both mouse strains. *Wt* infected mice reached their lowest weights around 8 dpi, while *Plscr1*^−/−^ infected mice experienced continued weight loss for an additional 2 days. *Plscr1*^−/−^ mice exhibited significantly greater weight loss compared to *Wt* mice with both sublethal and lethal dose infection at multiple timepoints (Figure 1C and 1D). Moreover, upon infection with 900pfu lethal dose of IAV, *Plscr1*^−/−^ mice had a significantly lower survival rate (Figure 1E). In fact, 100% of *Plscr1*^−/−^ mice died with 900 pfu of IAV in our experiment by 10 dpi.

To visualize IAV burden and spread from upper respiratory tract, paraffin-embedded lung sections were stained for H1N1-FITC. In contrast to *Wt* lungs where IAV infection was mostly local and restricted to major airways, *Plscr1*^−/−^ lungs had viral dissemination extending from bronchioles to alveoli, with ~50% of lungs infected. Moreover, *Plscr1*^−/−^ lungs had significantly higher corrected total cell fluorescence (CTCF) than *Wt* lungs at 3 dpi, suggesting an overall higher viral burden during acute infection in the absence of Plscr1 (Figure 1F). This observation was further supported by qRT-PCR analysis using cryopreserved lungs, as IAV M gene segment mRNA was significantly higher in *Plscr1*^−/−^ lungs at 3 dpi (Figure 1G).

### Plscr1 Limits Innate Immunity-Mediated Inflammation and Lung Damage in IAV Infection

**Figure 2.**
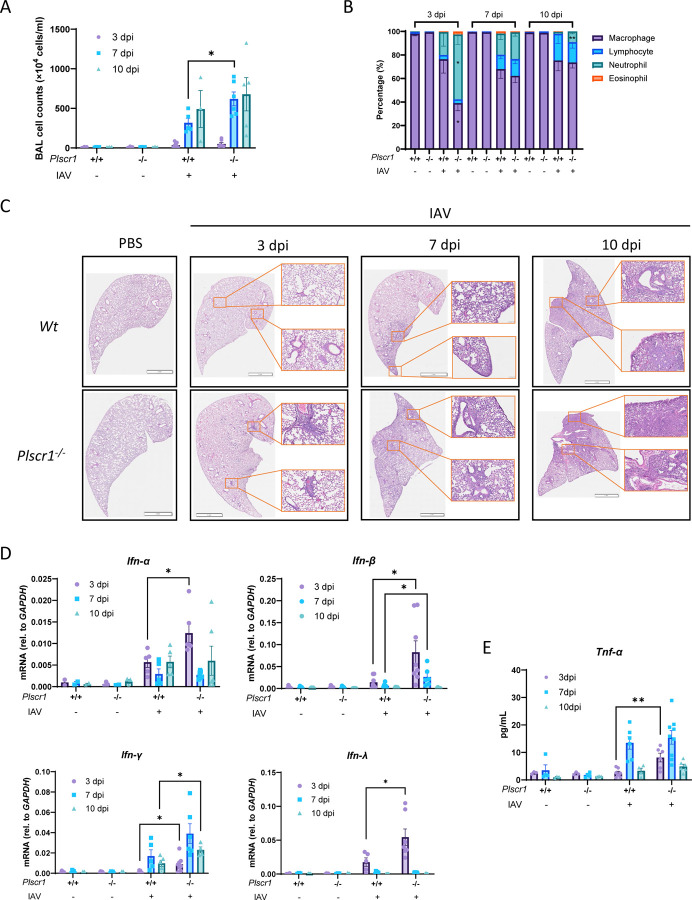
Increased Lung Inflammation in Plscr1−/− Mice in Influenza Virus Infection *Wt* and *Plscr1*^−/−^ mice were exposed to sublethal (300 pfu) IAV (WSN) infection. (A) Total BAL leukocyte numbers. (B) Differential cell counts in BAL. (C) Representative lung sections stained with H&E. Scale bars represent 3 mm (main) and 200 μm (inlays). (D) Whole lungs were analyzed for *Ifn-α*, *Ifn-β*, *Ifn-γ* and *Ifn-λ* RNA by qRT-PCR. (E) Tnf-α concentration in BAL by ELISA. Data are expressed as mean ± SEM of n = 5–10 mice/group. All data were pooled from three independent experiments. *p < 0.05, **p < 0.01. dpi, days post infection.

Bronchoalveolar lavage (BAL) was harvested for inflammatory cell counts using Cytospin followed by Diff-Quik stain. Immune cells infiltrated the lungs starting at 3 dpi and persisted at least until 10 dpi. *Plscr1*^−/−^ mice had significantly higher total BAL cell counts at 7 dpi compared to *Wt* mice, indicating a heightened inflammatory environment in alveoli during acute infection in the absence of Plscr1 (Figure 2A). IAV attracted neutrophils in early infection (3–7 dpi) and lymphocytes in late infection (7–10 dpi). Interestingly, *Plscr1*^−/−^ lungs were significantly more neutrophilic at 3 dpi, and neutrophil populations persisted until 10 dpi (Figure 2B).

Lung sections were stained with Hematoxylin and Eosin (H&E) and histopathology was assessed. *Plscr1*^−/−^ lungs showed minor and localized tissue damage as early as at 3 dpi, when *Wt* lungs appeared normal. Consistently, aggravated immunopathology was evident in *Plscr1*^−/−^ lungs at 7 and 10 dpi compared to *Wt* lungs (Figure 2C). These observations included increased thickening and collapse of alveolar walls, pulmonary edema surrounding alveolar walls, inflammatory cell infiltration in peri-bronchial and parenchymal areas, and hyperemia in the absence of Plscr1, suggesting its role in maintaining tissue homeostasis in IAV infection.

To assess the role of Plscr1 as an ISG, IFN expressions in whole lungs were measured by qRT-PCR. We found significantly elevated levels of *IFN-α*, *β*, *γ* and *λ* expression in *Plscr1*^−/−^ mice, particularly in early infection (Figure 2D). This upregulation was associated with heightened production of Tnf-α at 3 dpi, as assessed by ELISA (Figure 2E). These cytokines may implicate in hyperactive feedforward inflammatory circuits during early IAV infection leading to acute lung injury in *Plscr1*^−/−^ mice[21], consistent with worsened histopathology. On the contrary, the expressions of these mediators in *Wt* mice were well controlled, facilitating virus elimination without inciting excessive inflammation. These findings underscore the antiviral and potentially immunoregulatory role of Plscr1.

### Plscr1 Binds to *Ifn-λr1* Promoter and Activates *Ifn-λr1* Transcription in IAV Infection

**Figure 3.**
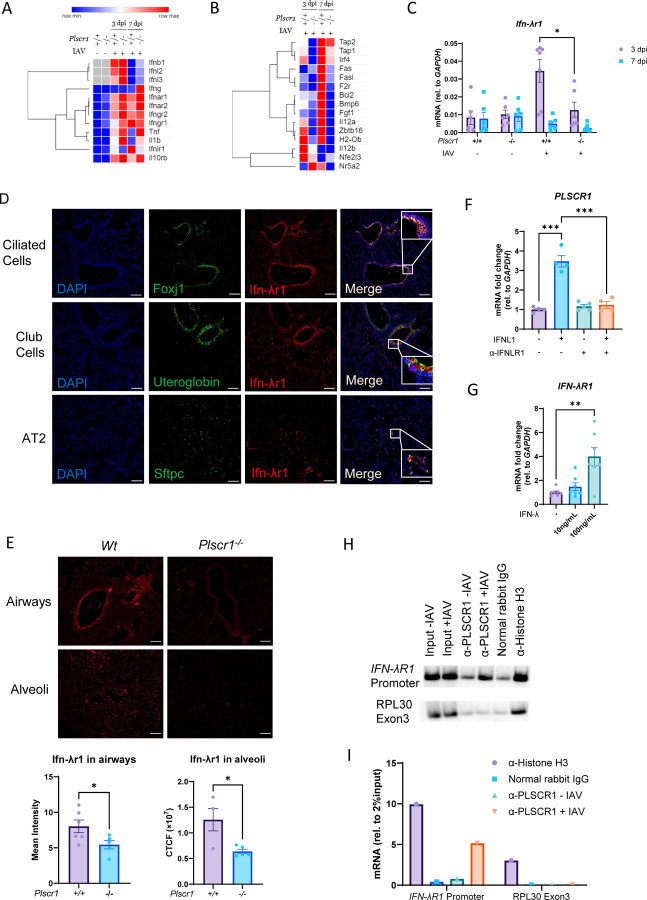
Transcriptional Regulation of IFN-λR1 by PLSCR1 and IFN-λ in IAV Infection (A-E) *Wt* and *Plscr1*^−/−^ mice were exposed to sublethal (300 pfu) IAV (WSN) infection. (A) Heatmap of interferons and their receptors in whole lungs by RNA-seq. (B) Heatmap of downregulated ISGs in whole lungs by RNA-seq. (C) Whole lungs were analyzed for *Ifn-λr1* by qRT-PCR. (D) Localization of Ifn-λr1+ cells in the lungs of IAV-infected *Wt* mice at 7 dpi. Sections stained for Ifn-λr1 (red), Foxj1, uteroglobin or Sftpc (green) and DAPI (blue) are shown. (E) Representative staining for Ifn-λr1 in airways or alveoli of IAV-infected *Wt* and *Plscr1*^−/−^ mice at 7dpi. Quantifications were performed using ImageJ. (F-G) Calu-3 cells were analyzed for *PLSCR1* (F) and *IFN-λR1* (G) RNA by qRT-PCR after recombinant IFN-λ and/or α-IFN-λR1 antibody treatment. Data are presented as fold change compared to non-treated group. (H-I) Chromatin-Immunoprecipitation of PLSCR1 and *IFN-λR1* promoter in Calu-3 cells followed by standard PCR (H) and real-time quantitative PCR (I). Data are expressed as mean ± SEM of n = 4–8 mice or wells/group. All data were pooled from three independent experiments. *p < 0.05, **p < 0.01, ***p<0.001. dpi, days post infection. CTCF, Corrected Total Cell Fluorescence. Scale bars represent 50 μm.

To determine if IFN signaling pathways are regulated by Plscr1 during IAV infection, RNA sequencing was performed using pooled samples from *Wt* and *Plscr1*^−/−^ mouse lungs infected with 300 pfu of IAV. A comprehensive examination of interferons and their receptors revealed that *Ifn-λr1* expression was significantly lower in *Plscr1*^−/−^ mice, despite high expression of *Ifn-λ* at both 3 and 7 dpi (Figure 3A). Importantly, *Ifn-λr1* was the only IFN receptor exhibiting this pattern, as the expression of its coreceptor *Il10-rβ* and other IFN receptors remained unaltered. The RNA sequencing result was further validated by qRT-PCR (Figure 3C). At a more fundamental level, we also observed alternations in ISG expressions, especially at 7 dpi. Many ISGs failed to be transcribed when *Ifn-λr1* function was impaired, including *Fas, FasL* and *Bcl2* (Figure 3B). As a result, disrupted type 3 interferon signaling due to impaired expression of *Ifn-λr1* may underlie the delayed viral clearance and exacerbated immunopathology observed in *Plscr1*^−/−^ mice[9].

To corroborate the cell-specific localization of Ifn-λr1 expression, *Wt* IAV-infected mouse lungs were probed with immunofluorescent antibodies to detect Ifn-λr1 and Foxj1 (a marker for ciliated epithelial cells), Uteroglobin (a marker for club cells) or Sftpc (a marker for type 2 alveolar epithelial (AT2) cells). Consistent with previous studies, Ifn-λr1 was present on all of these epithelial cell types (Figure 3D). We further quantified Ifn-λr1 expression in selected airways by measuring mean intensity to disregard the differences in airway areas. Ifn-λr1 expression in alveoli were measured using Corrected Total Cell Fluorescence (CTCF), so that all fluorescent signals within the same size of alveolar areas were analyzed. Expression of Ifn-λr1 was significantly reduced in *Plscr1*^−/−^ mouse lungs, in both airways and alveoli regions (Figure 3E). Calu-3 epithelial cells were then used to investigate if *PLSCR1* expression can be regulated by IFN-λ. Using qRT-PCR, we found *PLSCR1* transcription was significantly increased when stimulated with IFN-λ, and this effect was attenuated by pre-incubation with α-hIFNLR1 neutralizing antibody (Figure 3F). Additionally, IFN-λ could directly stimulate the expression of its receptor, *IFN-λR1*, thereby establishing a positive feedback loop to further drive the expression of *PLSCR1* (Figure 3G). These studies demonstrate that *PLSCR1* is an ISG that can be directly stimulated by IFN-λ in airway epithelial cells.

We subsequently investigated the mechanism underlying Plscr1’s transcriptional regulation of *Ifn-λr1* in airway epithelial cells. Chromatin-immunoprecipitation (ChIP) followed by standard PCR in IAV-infected Calu-3 cells unveiled that Plscr1 physically bound to the promoter region of *Ifn-λr1*, with this binding becoming more evident in IAV-infected cells (Figure 3H). These results were further validated using real-time quantitative PCR, suggesting that Plscr1 translocated into the nucleus of lung epithelial cells upon IAV infection to activate *Ifn-λr1* transcription (Figure 3I).

To further determine the regulation of Ifn-λr1 by Plscr1 in response to Ifn-λ signaling without the complexities associated with live virus infection, high molecular weight poly(I:C) was administered intranasally to mice daily for 6 days at 2.5mg/kg of body weight, and mice were sacrificed on the following day (Supplemental Figure 1A). We observed an increase in total BAL cell counts with poly(I:C) administration, although there was no difference between *Wt* and *Plscr1*^−/−^ mice (Supplemental Figure 1B). However, *Plscr1*^−/−^ mice exhibited a significantly higher number of lymphocytes in BAL (Supplemental Figure 1C), indicating that repetitive poly(I:C) administration might activate adaptive immune responses rather than the innate immune system in an acute IAV infection (Figure 1B). All interferon expressions were elevated following poly(I:C) administration, with comparable levels observed between *Wt* and *Plscr1*^−/−^ mice (Supplemental Figure 1D). Consistently, lung histopathology exhibited similar levels of inflammation or tissue damage in both *Wt* and *Plscr1*^−/−^ mice (Supplemental Figure 1E). Importantly, *Plscr1* expression was significantly induced by poly(I:C) (Supplemental Figure 1F). Notably, although no other phenotypical or genotypical variances were observed in poly(I:C)-treated *Plscr1*^−/−^ mice, *Ifn-λr1* expression was significantly lower in these mice, further affirming the requirement of Plscr1 for *Ifn-λr1* expression in response to IFN-λ (Supplemental Figure 1G).

### Plscr1 Interacts with Ifn-λr1 on Pulmonary Epithelial Cell Membrane in IAV Infection

**Figure 4.**
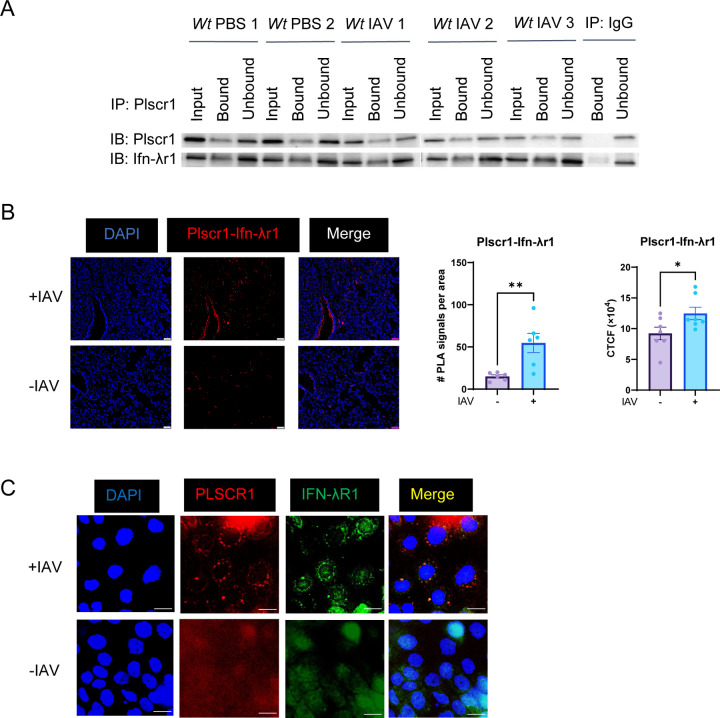
Protein Interaction between IFN-λR1 and PLSCR1 in IAV Infection (A) Co-Immunoprecipitation of Plscr1 and Ifn-λr1 in whole mouse lungs followed by western blot. (B) Proximity ligation assay of Ifn-λr1 and Plscr1 in the lungs of *Wt* mice infected or uninfected with IAV. Scale bars represent 50 μm. Quantifications were performed using ImageJ. (C) Colocalization of IFN-λR1 (green) and PLSCR1 (red) on Calu-3 cell membranes infected or uninfected with IAV in a nonpermeabilized staining. Scale bars represent 10 μm. Data are expressed as mean ± SEM of n = 6–7 lungs/group. All data were pooled from three independent experiments. *p < 0.05, **p < 0.01. PLA, Proximity Ligation Assay. CTCF, Corrected Total Cell Fluorescence.

We employed a combination of multiple biophysical approaches to fully assess the potential protein interaction between Plscr1 and Ifn-λr1. Co-immunoprecipitation (Co-IP) followed by western blot demonstrated that Plscr1 successfully pulled down Ifn-λr1 in both uninfected and IAV-infected *Wt* mouse lungs (Figure 4A). Consistently, using proximity ligation assay (PLA, Figure 4B), we detected direct interactions between Ifn-λr1 and Plscr1 on airway and alveolar epithelial cells in *Wt* mouse lungs. Noteworthily, significantly more and stronger PLA signals per area were detected in IAV-infected lungs compared to uninfected lungs. In agreement with the PLA, in unpermeablized Calu-3 cells, Plscr1 and Ifn-λr1 colocalized on the cell membrane after IAV infection (Figure 4C). In contrast, neither colocalization nor clear expression of Plscr1 or Ifn-λr1 was evident without infection, implying an infection-specific interaction in human airway epithelial cells (Figure 4C). Hence, Plscr1 may regulate Ifn-λr1 expression and IFN-λ signaling in airway epithelial cells through both gene transcription and protein interaction in IAV infection.

### Both Cell Surface and Nuclear PLSCR1 Regulates IFN-λ Signaling and Limits IAV Infection Independent of Its Enzymatic Activity

PLSCR1 mutants with specific PLSCR1 cellular distribution were employed to determine the relative contributions of cell surface and nuclear PLSCR1 in regulating IFN-λ signaling and IAV infection. PLSCR1 contains a 5-cysteine palmitoylation motif (C^184^CCPCC^189^), where substitution of these cysteines with alanine (labeled 5CA) completely abolishes its membrane localization, leading to exclusive localization in the cytosol and nucleus[22]. On the other hand, a single amino acid mutation of histidine^262^ to tyrosine (labeled H262Y) in the non-classical nuclear localization signal of PLSCR1 completely abates its nuclear localization, leaving PLSCR1 exclusively in the cytosol and on the cell membrane[23]. In addition, given that PS externalization plays a role in cell death regulation[14], we asked whether the enzymatic activity of PLSCR1 interferes with IFN-λ signaling. Phenylalanine^281^ in the β-barrel hydrophobic loop region of PLSCR1 has been shown to be important for Ca^2+^-dependent PS exposure[23], and a substitution with alanine (labeled F281A) renders PLSCR1 enzymatically inactive [24].

*PLSCR1(WT), PLSCR1(5CA), PLSCR1(H262Y)* and *PLSCR1(F281A)* plasmids built on PLV-EF1a-IRES-Hygro backbone were amplified in DH5α competent cells, co-transfected with HIV lentivector into 293T cells, and transduced into *Plscr1*^−/−^ A549 cells. After a 10-day hygromycin selection, transduction efficiency and subcellular locations of PLSCR1 were analyzed using flow cytometry (Supplemental Figure 2A). *PLSCR1(WT)* exhibited higher PLSCR1 expression than *Plscr1*^*+/+*^ cells, indicating overexpression resulting from transduction (Supplemental Figure 2B). Consistent with previous findings, *PLSCR1(5CA)* exhibited low surface expression, and *PLSCR1(H262Y)* had low nuclear expression of PLSCR1. *PLSCR1(F281A)* showed a similar distribution compared to *PLSCR1(WT)*, confirming that the loss of enzymatic activity did not restrict the subcellular localization. Transduction efficiency ranged between 37% to 70% (Supplemental Figure 2C).

**Figure 5.**
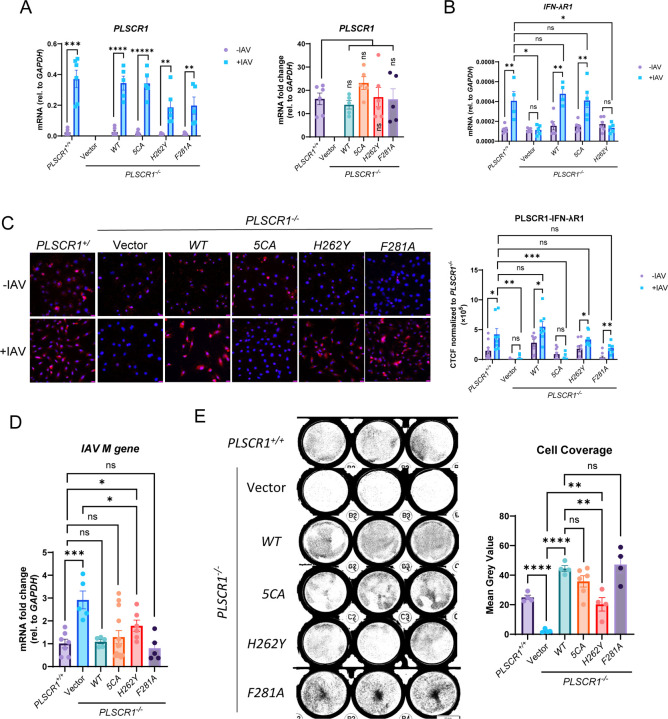
Requirement of Both Nuclear and Surface PLSCR1 but Not the Enzymatic Activity in IFN-λR1-Mediated Anti-Influenza Activities *PLSCR1*^−/−^ A549 cells were transduced with mutated *PLSCR1* plasmids using lentivirus and infected with IAV (PR8) for 24 hours at 1 MOI (A-D) or 10 MOI (E). (A) *PLSCR1* RNA by qRT-PCR. Data are also presented as fold change compared to uninfected groups. (B) *IFN-λR1* RNA by qRT-PCR. (C) Proximity ligation assay of IFN-λR1 and PLSCR1. Scale bars represent 20 μm. Quantifications were performed using ImageJ. (D) Viral RNA load was assessed by quantifying M gene by qRT-PCR. (E) Cells were stained with crystal violet. Cell viability was quantified using ImageJ. Data are expressed as mean ± SEM of n = 4–13 wells/group. All data were pooled from three independent experiments. ns, not significant, *p < 0.05, **p < 0.01, ***p<0.001, ****p<0.0001, *****p<0.00001. CTCF, Corrected Total Cell Fluorescence.

Upon IAV infection, *PLSCR1* was robustly induced in *PLSCR1*^*+/+*^ cells or cells transduced with all PLSCR1 constructs (Figure 5A), consistent with previous *in vivo* data (Figure 1G). In *PLSCR1*^*+/+*^ cells or cells transduced with *PLSCR1(WT)* or *PLSCR1(5CA)*, *IFN-λR1* expression was significantly induced by IAV infection. We found that PLSCR1 nuclear import was required for *IFN-λR1* transcription, as *IFN-λR1* transcription was not induced in cells transduced with *PLSCR1(H262Y)* or in *PLSCR1*^−/−^ cells (Figure 5B). In contrast, minimal protein interactions between PLSCR1 and IFN-λR1 were detected by PLA in cells transduced with *PLSCR1(5CA) or in PLSCR1*^−/−^ *cells,* while *PLSCR1*^*+/+*^ cells and cells transduced with *PLSCR1(WT)*, *PLSCR1(H262Y)* and *PLSCR1(F281A)* had strong protein binding of PLSCR1-IFN-λR1, demonstrating that PLSCR1 interacts with IFN-λR1 on cell membrane (Figure 5C). Furthermore, the lipid scramblase activity of PLSCR1 is uncoupled from its regulation of IFN-λ signaling.

When compared to *PLSCR1*^*+/+*^ cells, *PLSCR1(WT)*, *PLSCR1(5CA)* and *PLSCR1(F281A)* similarly protected against IAV infection, but *PLSCR1(H262Y)* only exhibited a partial viral clearance, shown by viral copy numbers extracted from cells (Figure 5D). This is further validated with a cell coverage assay when a high dose IAV was used for infection. Since the proliferation between differently transduced cell lines was similar and all cells were grown to full confluency before infection, the cell coverage represented resistance to infection and survival of cells. While *PLSCR1(5CA)* and *PLSCR1(F281A)* had a high surface coverage comparable to *PLSCR1(WT)*, *PLSCR1(H262Y)* lost about half of the coverage (Figure 5E). Therefore, nuclear PLSCR1 is both essential and sufficient for IAV control through *IFN-λR1* transcription regulation. Nevertheless, *PLSCR1(H262Y)* still rescued a significant number of cells when reintroduced into *PLSCR1*^−/−^ cells, indicating a partial protective role of PLSCR1 on epithelial cell surface through IFN-λR1 protein regulation. Moreover, the anti-IAV function of PLSCR1 is independent of its lipid scramblase activity.

### PLSCR1 Expression Is Upregulated in the Ciliated Airway Epithelial Compartment of Mice following Flu Infection

**Figure 6.**
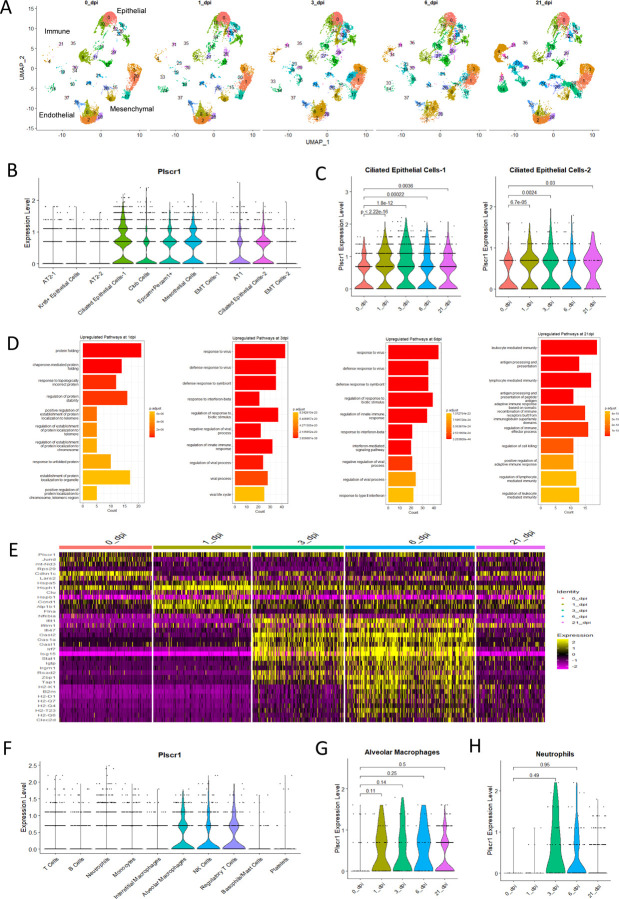
Cell-Specific Roles of Plscr1 in Influenza Virus Infection in Mice *Wt* mice were exposed to 2500 EID50 IAV (PR8) infection. Lungs were used for single-cell RNA sequencing analysis at 0, 1, 3, 6 and 21 dpi. (A) Two-dimensional UMAP representation of individual cells obtained from different timepoints. (B) Aggregated *Plscr1* expressions in all epithelial cell clusters. (C) Time-dependent *Plscr1* expressions in both ciliated epithelial cell clusters. (D) GO analysis for upregulated pathways in Ciliated Epithelial Cells-1. (E) Heatmap of the most differentially expressed genes of Ciliated Epithelial Cells-1 at different timepoints. (F) Aggregated *Plscr1* expressions in all immune cell clusters. (G) *Plscr1* expressions in alveolar macrophage cluster. (H) *Plscr1* expressions in neutrophil cluster.

To understand the specific cell types that have increased PLSCR1 expression following flu infection *in vivo*, we explored a comprehensive scRNA sequencing dataset generated from uninfected or IAV-infected mouse lungs at 0, 1, 3, 6 and 21 dpi[25]. Distinct lung cell populations were identified using the Seurat algorithm. We assigned a total of 38 clusters based on their transcriptomic signatures, including 13 epithelial populations, 5 endothelial populations, 9 mesenchymal populations and 11 immune populations (Table S2). Two-dimensional UMAP demonstrated that these main lung cell populations (epithelial, endothelial, mesenchymal, and immune) were dynamic over the course of infection (Figure 6A).

Within the epithelial populations, *Plscr1* was mainly expressed by ciliated epithelial cells, mesothelial epithelial cells, Epcam+Pecam1+ cells, club cells and AT1 cells (in decreasing order of aggregated expression, Figure 6B). Conversely, AT2 cells, epithelial-mesenchymal transitional cells and Krt8+ cells exhibited very low levels of *Plscr1* expression. Among the various epithelial populations, ciliated epithelial cells not only had the highest aggregated expression of *Plscr1*, but also were the only epithelial cell population in which significantly more *Plscr1* was induced in response to IAV infection. Moreover, the increased expression of Plscr1 persisted for at least 21 days in ciliated epithelial cells, suggesting that Plscr1 may primarily exert its anti-flu activities in these cells (Figure 6C).

For the predominant ciliated epithelial cell cluster, we performed Gene Ontology (GO) analysis of the differentially expressed genes to compare potentially distinct functions before and after infection (Figure 6D). We found that the most upregulated pathways at 1 dpi were related to protein folding and localization, indicating a rapid cellular response to infection by altering their protein profile. At 21 dpi, ciliated epithelial cells were characterized by adaptive immune regulation, such as antigen presentation and memory cell generation. At 3 and 6 dpi, ciliated epithelial cells exhibited similar innate immune and inflammatory signatures dominated by interferon signaling. As expected, *Plscr1* participated in all of the top 10 enriched pathways, implying that it could facilitate robust antiviral responses in ciliated epithelial cells as an ISG during acute infection.

Gene expression levels of the most differentially expressed genes at individual timepoints in ciliated epithelial cells were shown in a heatmap (Figure 6E). Signature genes for cells at homeostasis included *Jund* (JunD proto-oncogene), *mt-Nd3* (mitochondrial NADH dehydrogenase 3) and *Cdkn1c* (cyclin-dependent kinase inhibitor 1C), which are regulators of cell metabolism and proliferation[26–28]. Signature genes enriched at 1 dpi were *Hspa5* (heat shock protein family A member 5), *Hsph1* (heat shock protein family H member 1) and *Clu* (clusterin), which play critical roles in protein folding and assembly[29–31]. Consistent with the GO analysis, ciliated cells at 3 and 6 dpi had very similar signature genes associated with interferon pathways, namely a large component of ISGs, such as *Isg15* (interferon-stimulated gene 15), *Oasl2* (2'-5'-oligoadenylate synthetase like 2) and *Irf7* (interferon regulatory factor 7). Importantly, Plscr1 is required to potentiate the expressions of many of these ISGs[20], highlighting its potentially multifunction in anti-flu responses of ciliated epithelial cells. Signature genes for 21 dpi largely overlapped with those for 10 dpi, containing many histocompatibility 2, class II antigen proteins, which are mediators of MHC class II antigen processing and presentation.

In contrast to epithelial clusters, immune populations had low levels of *PLSCR1*, with relatively high expressions observed in alveolar macrophages, NK cells and regulatory T cells (Figure 6F). However, we did not observe any significant difference in PLSCR1 levels before and post-infection in macrophages and neutrophils (Figure 6G and 6H), the two immune populations that express IFN-λR1 besides dendritic cells[32]. Hence, the anti-influenza activities we described are unlikely to be carried out by immune cells.

### Overexpression of Plscr1 in Ciliated Epithelial Cell, but Not Myeloid Cell, is Sufficient to Provide Anti-Flu Protection through IFN-λ Signaling

**Figure 7.**
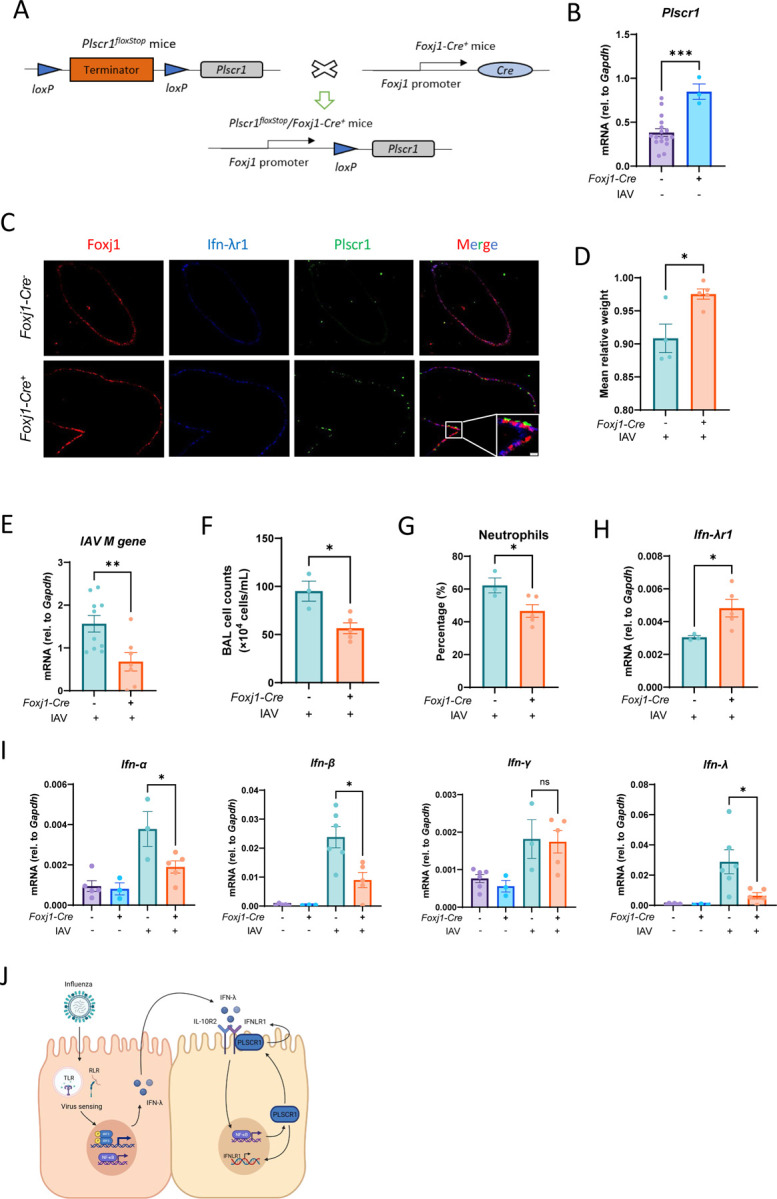
Reduced Susceptibility of Plscr1floxStopFoxj1-Cre+ Mice to Influenza Virus Infection *Plscr1*^*floxStop*^ and *Plscr1*^*floxStop*^*Foxj1-Cre*^*+*^ mice were exposed to sublethal (300 pfu) IAV (WSN) infection and sacrificed at 3 dpi. (A) Generation of ciliated epithelial cell conditional *Plscr1 KI* mice. (B) Validation of *Plscr1* overexpression in lungs of *Plscr1*^*floxStop*^*Foxj1-Cre*^*+*^ mice by qRT-PCR. (C) Representative immunofluorescent staining for Plscr1, Ifn-λr1 and Foxj1 in lungs. (D) Mean relative weight of mice. (E) Viral RNA load in the lungs was assessed by quantifying M gene by qRT-PCR. (F) Total BAL leukocyte numbers. (G) Neutrophil percentages in BAL. (H) Whole lungs were analyzed for *Ifn-λr1* RNA by qRT-PCR. (I) Whole lungs were analyzed for *Ifn-α*, *Ifn-β*, *Ifn-γ*, *Ifn-λ* RNA by qRT-PCR. (J) Model depicting proposed mechanism of PLSCR1-regulated IFN-λ signaling Data are expressed as mean ± SEM of n= 3–10 mice/group. ns, not significant, *p < 0.05, **p < 0.01, ***p<0.001. dpi, days post infection.

To further validate the relevant contributions of ciliated epithelial cells versus myeloid populations including macrophages and neutrophils, we generated *Rosa26* locus targeted *Plscr1* conditional knock-in transgenic mice (*Rosa26-LoxP-STOP-LoxP-Plscr1 Tg*; labeled *Plscr1*^*floxStop*^). These mice were bred with *Foxj1-Cre* or *LysM-Cre* mice to overexpress Plscr1 in ciliated epithelial cells or myeloid cells respectively (Figure 7A). Overexpression was confirmed by qRT-PCR with whole lung lysate and immunofluorescence staining at baseline (Figure 7B, 7C and Supplemental Figure 3A).

At 3 dpi with sublethal IAV infection, *Plscr1*^*floxStop*^*Foxj1-Cre*^*+*^ mice lost less body weight (Figure 7D), and had lower viral copy numbers (Figure 7E) compared to *Plscr1*^*floxStop*^ mice, indicating overexpression of Plscr1 in ciliated epithelial cells provides protection against flu infection. Concurrently, they had significantly lower total BAL cell counts (Figure 7F), lower neutrophil percentages (Figure 7G) and lower type 1 and 3 *Ifn* expressions (Figure 7I). We then examined Ifn-λr1, and found that its transcription was further increased in *Plscr1*^*floxStop*^*Foxj1-Cre*^*+*^ mice during acute infection (Figure 7H). Therefore, ciliated epithelial cell-specific overexpression of Plscr1 is sufficient to provide anti-influenza protection through IFN-λ signaling. In contrast, using similar approaches, we found that overexpression of Plscr1 in myeloid cells did not protect mice from IAV infection: *Plscr1*^*floxStop*^*LysM-Cre*^*+*^ mice exhibited comparable weight loss, total BAL cell counts, viral copy numbers, histopathology and expressions of interferons, *Ifn-λr1* and *Plscr1* at both early and late timepoints examined (Supplemental Figure 3B-3I). Taken together, consistent with the scRNA sequencing, transgenic mouse models with cell-specific overexpression of Plscr1 demonstrated that Plscr1 offers antiviral protection in ciliated airway epithelial cells through IFN-λ signaling.

## DISCUSSION

We are the first group to demonstrate the roles of Plscr1 in a mouse-adapted human IAV-infected mouse model, to implicate its IFN-λ signaling-related mechanisms, and to elucidate the cell types that are responsible for Plscr1-mediated anti-influenza activities. We established *Plscr1*^−/−^ mice and found them more susceptible to IAV (WSN) compared to *WT* mice, as evidenced by greater weight loss in both sublethal and lethal infection and poorer survival in a lethal infection. Further examination of infected lungs provided the first *in vivo* evidence demonstrating that Plscr1 suppressed human IAV replication. This observation aligns with previous report indicating that PLSCR1 interacts with the IAV NP, thereby impairing its nuclear import *in vitro*[17]. Notably, while differences in viral copy numbers were only observed at the early stages of infection, coinciding with a significant increase in *Plscr1* transcription, these changes had profound implications for host fitness. Therefore, as one of the earliest induced ISGs, Plscr1 constitutes the frontline defense against influenza infection.

While the only previously published *Plscr1*^−/−^ mouse flu model focused on an H1N1 SIV infection[18], our data showed both similarities and discrepancies. First, while both studies observed that Plscr1 promoted survival during IAV infection, SIV-infected *Plscr1*^−/−^ mice exhibited weight loss similar to *Wt* mice. Furthermore, while both models attributed the lower survival rate in *Plscr1*^−/−^ mice to increased viral replication, SIV-infected *Plscr1*^−/−^ lungs exhibited higher viral titers across all examined time points, from 1 to 7 dpi. Intriguingly, contrary to our observations, *Plscr1* expression was markedly decreased in SIV infection. Given previous *in vitro* studies demonstrating PLSCR1 induction by IAV (WSN)[17] and type 1 IFNs[15, 20, 33], we propose that the contradictory trend observed by Liu et al. may be attributed to distinct properties of SIV, such as viral replication rate, both the cellular tropism and the tissue tropism (proximal or distal lung), or antigen variation which may affect direct interaction with PLSCR1, innate sensing of the infection, or recognition by the adaptive immune response.

The delicate balance between immunity and immunopathology plays a pivotal role in determining host fitness during viral infections. To interrogate immunopathology in the lungs, we accessed the BAL, histology and interferon expressions. BAL from *Plscr1*^−/−^ mice were highly enriched with inflammatory neutrophils and lymphocytes, which were likely attracted by robust IFNs and other chemokines. Consistently, *Plscr1*^−/−^ mice exhibited more severe lung damage and a greater extent of affected areas. These findings indicate that Plscr1 not only enhances immunity but also mitigates immunopathology. Importantly, regardless of excessive production of antiviral IFNs in *Plscr1*^−/−^ mice, they failed to effectively control the initial viral infection. This suggests that the absence of Plscr1 impairs the IFN signaling pathway, highlighting the crucial role of Plscr1 in facilitating effective antiviral responses.

Although type 1 and 3 IFNs may share similar downstream pathways, they rely on distinct receptors for signaling. Consistent with previous findings[3], Ifn-λr1 was detected in respiratory epithelium, including ciliated epithelial cells, club cells and AT2 cells during infection. Loss of Plscr1 exclusively impaired *Ifn-λr1* transcription in early IAV infection, with this transcriptional difference translating into protein expression. IFN-λ is crucial for early viral control within the initial days of infection without igniting unnecessary inflammation and compromising host fitness[9]. With limited Ifn-λr1 expression, *Plscr1*^−/−^ mice were unable to mount a robust type 3 IFN response to control early viral infection. Instead, they relied largely on type 1 interferons, which succeeded in eliminating IAV at later time points, but led to exaggerated immunopathology. Furthermore, our observations of enhanced neutrophilia, lung injury and lethality in *Plscr1*^−/−^ mice align with findings reported in *Ifn-λr1*^−/−^ mice in IAV infection [9].

PLSCR1 expression was increased in response to IFN-λ in human airway epithelial cells, consistent with previous studies[24]. While PLSCR1 typically localizes on the cell membrane and in the cytoplasm, it translocates into nucleus to bind the *IFN-λR1* promoter upon IAV infection, thereby regulating *IFN-λR1* transcription. The nuclear localization and functions of PLSCR1 have been extensively documented in previous studies[12, 34–36]. Relevantly, IFN-α promotes the nuclear translocation of PLSCR1 in breast cancer cells[22]. Therefore, it is highly plausible that the nuclear trafficking of PLSCR1 in airway epithelial cells is similarly stimulated by IFN-α produced during IAV infection, but further evidence is demanded. Additionally, the precise binding site for PLSCR1 within the *IFN-λR1* promoter and the binding motif on PLSCR1 remain unknown. Previous bioinformatics predictions revealed that the −430~−421 segment of *IFN-λR1* promoter likely contains binding sites for a number of transcription factors (TFs) with important regulatory functions[37]. Further mutagenesis studies, such as truncations or single nucleotide mutations within these sequences, could be done to identify the specific motif for PLSCR1 binding. Finally, it is not clear whether PLSCR1 directly activates *IFN-λR1* expression by acting as a TF, or as a co-factor enhancing other TF’s transcriptional activity. Coimmunoprecipitations could be pursued in future to explore the binding potential between PLSCR1 and other known TFs for *IFN-λR1*, such as NF-Y[37].

In addition to activities in the nucleus, PLSCR1 has been shown to interact with multiple proteins on the plasma or endosomal membrane[19, 38–41]. Here we reported a novel interaction between PLSCR1 and IFN-λR1 on airway epithelial cell membrane *in vivo* and *in vitro*, confirmed with both coimmunoprecipitation and immunofluorescence. Since their interaction was significantly enhanced after IAV infection, we speculate that membrane-bound PLSCR1 is a positive regulator of IFN-λR1 signaling. One plausible mechanism is that PLSCR1 facilitates the intracellular trafficking of IFN-λR1, akin to its role in assisting the trafficking of other membrane receptors[19, 39, 42].

The subcellular location of PLSCR1 is vital not only for interactions with host components, but also for direct viral control. We found that nuclear PLSCR1 is both necessary and sufficient for viral control in airway epithelial cells, whereas membrane PLSCR1 provides only partial protection against IAV infection. These findings are not surprising, as the previously reported anti-flu mechanism of PLSCR1 also relies on its nuclear localization signal to restrict the import of IAV NP[17]. Furthermore, besides *IFN-λR1*, PLSCR1 enhances the expression of a select subset of ISGs in IAV infection as well, a mechanism potentially mediated by nuclear PLSCR1 on ISG gene transcription[20]. On the other hand, membrane PLSCR1 may modulate the JAK/STAT signaling pathway, thereby augmenting the optimal anti-viral activity of these ISGs[20]. We found that membrane PLSCR1 interacts with IFN-λR1 protein in IAV infection, suggesting that it could facilitate viral elimination to some extent.

PLSCR1 is most well-known for its scramblase activity that favors PS exposure, apoptosis and phagocytosis[38, 43]. Using an enzymatically inactive mutant of PLSCR1, we uncoupled its lipid scramblase activity from anti-influenza activity. There are several potential explanations for this finding. First, our epithelial cell culture lacked phagocytes, therefore the impact of apoptosis followed by phagocytosis induced by PLSCR1 is minimal. Future studies using mice that harbor *Plscr1(F281A)* mutation would be needed to verify the role of lipid scramblase activity and epithelial cell apoptosis in the presence of phagocytes. Second, PLSCR1 exhibits only weak enzymatic activities compared to other members of lipid scramblase family, possibly due to its vastly different central β-barrel structure[24, 44]. PS externalization may be compensated by other more potent scramblases. Importantly, the lipid scramblase activity of PLSCR1 has been shown to be dispensable for its anti-SARS-CoV-2 function in a similar manner[24], suggesting a general lack of significance for its enzymatic activity in viral infections.

While scRNA-seq analysis revealed that endothelial cells express Plscr1 most abundantly in the lung, they are not the major target of IAV infection, and IAV does not efficiently replicate in them[45]. Instead, airway epithelial cells are the frontline defense against respiratory pathogens, with ciliated epithelial cells being the only cell type that express α2,3-linked SA, the primary influenza virus receptor in the mouse airway[46]. Coincidently, our scRNA-seq results showed that ciliated epithelial cells not only had the highest aggregated expression of *Plscr1*, but also were the only epithelial cell population in which significantly more *Plscr1* was induced in response to IAV infection. Experiments with *Plscr1*^*floxStop*^*Foxj1-Cre*^*+*^ mice further supported ciliated epithelial cell-dependent protection against IAV, with improved immunity and viral clearance, and dampened immunopathology at as early as 3 dpi. These findings suggest that as a result of enhanced Ifn-λr1 due to Plscr1 overexpression, type 3 interferons were able to exert their advantages being the earliest produced interferon, mounting both antiviral and anti-inflammatory responses in ciliated epithelial cells.

Taken together, our findings highlight the essential role of PLSCR1 in the regulation of IFN-λR1 transcription in nucleus and expression on plasma membrane, both *in vitro* and *in vivo*. These mechanisms are crucial for inhibiting viral spread, reducing inflammation, and enhancing overall host fitness during IAV infection. Further, we found that the enzymatic activity of PLSCR1 is dispensable for its anti-influenza function. Finally, ciliated airway epithelial cells are the primary cell type in the lung for mounting PLSCR1-mediated anti-influenza responses. The potential of PLSCR1 agonists that target ciliated airway epithelial cells as therapeutic treatments for influenza holds promise for future medical interventions. Moreover, our results have the potential to impact the classical yet evolving field of IFN signaling. Not only do these findings elucidate and expand our understanding of newly discovered IFN-λ signaling, but they also shed light on the specific cell types and conditions under which IFN-λ signaling is modulated. Given the significance of IFN-λ signaling in various infectious diseases, these insights may pave the way for innovative therapeutics approaches targeting corresponding regulatory molecules in the treatment of other microbial infections in addition to influenza.

## MATERIALS AND METHODS

### Viruses

Purified A/WSN/1933 and A/PR/8/1934 (H1N1) virus (IAV) was kindly provided by Dr. Amanda Jamieson, Brown University. MDCK cells (ATCC) were used for the preparation of virus stocks and for virus titration by plaque assay.

### Cell culture

Calu-3 cells were kindly provided by Dr. Suchitra Kamle, Brown University. Cells were maintained in Eagle’s Minimum Essential Medium (ATCC) with 10% fetal bovine serum (Gibco) and 1% penicillin-streptomycin (Sigma-Aldrich).

*PLSCR1*^−/−^ A549 cells were kindly provided by Dr. John MacMicking, Yale University. Cells were maintained in Dulbecco's Modified Eagle Medium (Genesee) with 10% fetal bovine serum, 1% MEM non-essential amino acids (Gibco) and 1mM sodium pyruvate (Gibco).

### Mice

Wild-type (WT) mice on a C57BL/6J genetic background were purchased from the Jackson Laboratories. *Plscr1*^*tm1a(EUCOMM)Hmgu*^ mice (*Plscr1*^−/−^ mice on C57BL/6 background) were purchased from the International Mouse Phenotyping Consortium. Those mice have an FRT flanked *lacZ*/neomycin sequence-tagged *LoxP* insertion upstream of *Plscr1* exon 6 and 7, and another *LoxP* insertion downstream of these critical exons. Subsequent *Cre* expression results in whole-body knockout mice that transcribed a shortened and nonfunctional transcript of *Plscr1*[47]. Genetic screening of *Plscr1*^−/−^ mice was carried out by conventional PCR on genomic DNA from mouse tail biopsies using the following primers for *LacZ* reporter: Fw: 5’-GCGATCGTAATCACCCGAGT-3’ and Rev: 5’-CCGCCAAGACTGTTACCCAT-3’. This set of primers generates a 307bp fragment in *Plscr1*^−/−^ mice.

*Rosa26* locus targeted *Plscr1* conditional knock-in transgenic mice (*Rosa26-LoxP-STOP-LoxP-Plscr1 Tg*; *Plscr1*^*floxStop*^ mice on C57BL/6 background) were generated at Brown Mouse Transgenic and Gene Targeting Facility. They were bred with *LysM-Cre* mice purchased from the Jackson Laboratories and *Foxj1-Cre* mice gifted by Drs. Yong Zhang and Michael Holtzman at Washington University School of Medicine in St. Louis to generate *Plscr1*^*floxStop*^*LysM-Cre*^*+*^ and *Plscr1*^*floxStop*^*Foxj1-Cre*^*+*^ mice respectively. For the detection of *Cre* recombinase in *LysM-Cre* mice, the following primers were used: Common Fw: 5’-CTTGGGCTGCCAGAATTTCTC-3’, Mutant Rev: 5’-CCCAGAAATGCCAGATTACG-3’ and Wild type Rev: 5’-TTACAGTCGGCCAGGCTGAC-3’. A 700bp fragment is amplified in homozygous mutants, while a 350bp fragment is amplified in wild type mice. Both fragments show up in heterozygotes. For the detection of *Cre* recombinase in *Foxj1-Cre* mice, the following primers were used: Mutant Fw: 5’-CGTATAGCCGAAATTGCCAGG-3’ and Mutant Rev: 5’-CTGACCAGAGTCATCCTTAGC-3’. A 327bp fragment is amplified in homozygous mutants.

All mice were housed and further bred in Brown University animal facilities. The mice with null mutation or overexpression of Plscr1 did not show any apparent abnormal phenotypes and developmental and signaling issues. All murine procedures were approved by the Institutional Animal Care and Use Committees at Brown University.

### Infection and treatment of mice

10–12-week-old mice were intranasally infected once with various doses of IAV in 30 μL of sterile PBS, or treated with 2.5 μg/g of body weight of poly(I:C) (Invivogen) every day for 6 days under isoflurane-based anesthesia. Poly(I:C) was warmed up in a water bath at 37 °C before administration. 30 μL of sterile PBS was administrated in control mice. Only males were used in infections. Littermates were randomly assigned to experimental and control groups.

### BAL harvest and differential cell counts

Mice were euthanized with intra-peritoneal injection of 300 μL of urethane (0.18 g/ml, Sigma-Aldrich). Bronchoalveolar lavage (BAL) of the whole lung was performed with a total of 1 mL ice-cold PBS via an incision at the trachea. An aliquot was stained with trypan blue solution (Gibco) for viability and cell number determination using TC20 automated cell counter (Bio-Rad). Samples were centrifuged at 2500 rpm for 5 min at 4°C. Cells were pelleted onto glass slides via cytospin centrifugation at 700 rpm for 7 min and stained with Hema 3 (Fisher Scientific). Neutrophils, macrophages, lymphocytes and eosinophils were counted under a light microscope.

### RNA isolation and qPCR

Harvested right lungs were immediately snap-frozen in liquid nitrogen and stored in 80°C afterwards. Frozen lungs were homogenized in TRIzol Reagent (Invitrogen). Calu-3 cells were washed with ice cold PBS once, directly lysed in the culture dish with TRIzol Reagent and collected using a cell scraper. RNA isolation was performed with RNeasy Mini Kit (Qiagen). 0.5 μg of the isolated RNA was used for cDNA synthesis with iScript cDNA Synthesis Kit (Bio-Rad). Real-time quantitative PCR was performed with iTaq Universal SYBR^®^ Green Supermix (Bio-Rad). All primers used were listed in Table S1. Relative amounts of mRNA expression were normalized to the level of mouse *Gapdh*.

### Immunohistology and immunofluorescence

For immunohistology, immediately after euthanasia, left lungs were inflated through the incision at the trachea with 10% buffered formalin and soaked in formalin at RT. They were then transferred to 70% ethanol prior to paraffin embedding, sectioning and staining with hematoxylin and eosin (H&E) performed at Brown University Molecular Pathology Core. Lungs were scanned with a VS200 slide scanner (Evident).

For immunofluorescence of mouse lungs, unstained paraffin-embedded lung sections were rehydrated in xylene (Fisher Scientific) and decreasing concentrations of ethanol (Pharmco). They were then steamed in antigen retrieval buffer (Abcam) for 30 minutes and blocked with 1% normal goat serum (Abcam) for 30 minutes at RT. Tissues were stained overnight at 4°C with goat anti-mouse Ifn-λr1 polyclonal antibody (1:300 dilution, Invitrogen), rabbit anti-mouse uteroglobin polyclonal antibody (1:300 dilution, Invitrogen), rabbit anti-mouse Spc polyclonal antibody (1:100 dilution, Santa Cruz Biotechnology), and/or mouse anti-mouse Foxj1 monoclonal antibody (1:300 dilution, Invitrogen). Tissues were washed 3 times with PBS and stained with chicken anti-goat Alexa 594 (Invitrogen), goat anti-rabbit Alexa 488 (Invitrogen) and/or donkey anti-goat Alexa 405 (Invitrogen) for 1 hour at RT in the dark. For visualization of IAV, tissues were stained overnight at 4°C with goat anti-H1N1-FITC (1:500 dilution, US Biological). After washing 3 times with PBS, sections were mounted with Vectashield^®^ mounting medium with DAPI (Vector Laboratories). Lungs were visualized under an APEXVIEW APX100 microscope (Evident).

For immunofluorescence of cell cultures, cells were seeded in Millicell EZ slides (Millipore Sigma). After washing with ice-cold PBS, 4% paraformaldehyde (Boston BioProducts) was added on ice for 10 minutes for fixation and removed. If necessary, 0.2% Triton X-100 (Sigma-Aldrich) was added for 10 minutes to permeabilize the cells on ice, followed by 3 times of PBS wash. Cells were blocked with 5% bovine serum albumin (Fisher Scientifics) for 1 hour at RT. They were stained overnight at 4°C with mouse anti-human PLSCR1 monoclonal antibody (1:50 dilution, R&D Systems) and rabbit anti-human IFN-λR1 polyclonal antibody (1:100 dilution, Invitrogen). Cells were washed 3 times with PBS and stained with chicken anti-mouse Alexa 594 (Invitrogen) and goat anti-rabbit Alexa 488 for 1 hour at RT in the dark. After washing 3 times with PBS, slides were mounted with Vectashield^®^ mounting medium with DAPI and visualized under a Nikon ECLIPSE Ti microscope.

When necessary, fluorescent images were analyzed using ImageJ[48]. Areas of interest were selected using freehand selection. Area, mean grey value and integrated density were measured. For H1N1 staining, uninfected lungs were used as background readings. For Ifn-λr1 staining, a region without fluorescence was used as a background reading. Corrected total cell fluorescence (CTCF) was calculated using the following formula: CTCF = Integrated Density – (Area of selection X Mean fluorescence of background readings).

### Proximity Ligation Assay (PLA)

Duolink^®^ PLA fluorescence (Millipore Sigma) was performed according to manufacturer’s protocol. In brief, unstained paraffin-embedded lung sections were rehydrated, retrieved and blocked with Duolink^®^ Blocking Solution for 60 minutes at 37°C. They were then incubated with goat anti-mouse Ifn-λr1 polyclonal antibody and rabbit anti-mouse Plscr1 polyclonal antibody (1:300 dilution, Proteintech) overnight at 4°C. Next, slides were incubated with anti-goat PLUS and anti-rabbit MINUS probes for 1 hour at 37°C. For cell culture, A549 cells were seeded in Millicell EZ slides, fixed and permeabilized. After blocking, cells were incubated with rabbit anti-human IFN-λR1 polyclonal antibody and mouse anti-human PLSCR1 monoclonal antibody overnight at 4°C. Next, cells were incubated with anti-mouse PLUS and anti-rabbit MINUS probes for 1 hour at 37°C. Diluted ligase and polymerase were applied to samples for 30 minutes at 37°C in order. Finally, slides were mounted with Duolink^®^ In Situ Mounting Medium with DAPI and imaged with an APEXVIEW APX100 microscope.

### IAV Infection and rhIFN-λ1 treatment in cell culture

Calu-3 cells at 80–90% confluency were washed twice with Opti-MEM (Gibco) and infected with IAV (WSN) at 0.1 multiplicity of infection (MOI) in Opti-MEM. One hour after viral attachment at 4°C, IAV supernatant was removed. Cells were washed once and incubated in fresh Opti-MEM for 23 hours at 37°C.

A549 cells at 80–90% confluency were washed once with PBS and infected with IAV (PR8) at 1, 5 or 10 MOI in PBS supplemented with BSA, CaCl_2_ and MgCl_2_. One hour after viral attainment at 37°C, IAV supernatant was removed. Cells were incubated in Opti-MEM with 10 μg/mL trypsin for 23 hours at 37°C.

Calu-3 or A549 cells were treated with 10 or 100 ng/mL of recombinant human IL-29/IFN-lambda 1 Protein (R&D Systems) for 6 or 24 hours at 37°C. In control Calu-3 groups, cells were first incubated with 1 μg/mL of anti-human interferon lambda receptor 1 neutralizing antibody (PBL Assay Science) for 1 hour, and then treated with IFN-λ.

### Co-immunoprecipitation (Co-IP) and western blot

Cryopreserved mouse lungs were homogenized in Pierce RIPA buffer (Thermo Scientific). Catch and Release^®^ v2.0 Reversible Immunoprecipitation System (Millipore) was used according to manufacturer’s protocol. In brief, 500 μg of lung lysate, 2 μg of rabbit anti-human Plscr1 polyclonal antibody or negative control human IgG and 10 μL of antibody capture affinity ligand were added to the spin columns with resin slurry. The mixture was incubated on a rotator at 4°C overnight. The unbound flow-through was collected by centrifuge. Bound proteins were washed 3 times and eluted using denaturing elution buffer (5% SDS, 8M Urea, and 100mM Glycine).

Twenty μg of each sample was loaded into 4–20% Mini-PROTEAN TGX Gels (Bio-Rad) and run for 1 hour 20 minutes at 125V in Tris/Glycine/SDS running buffer (Bio-Rad). Gels were transferred using Trans-Blot Turbo Transfer Pack (Bio-Rad). Membranes were incubated with either rabbit anti-mouse Plscr1 polyclonal antibody (1:1000 dilution) or rabbit anti-mouse Ifn-λr1 polyclonal antibody (1:1000 dilution, ABclonal) overnight at 4°C. After washing with TBST (Boston BioProducts), membranes were incubated with HRP-linked anti-rabbit IgG (1:5000 dilution, Cell Signaling) for 1.5 hour at RT. Finally, membranes were treated with SuperSignal West Pico PLUS Chemiluminescent Substrate (Thermo Scientific) and imaged with ChemiDoc Imaging Systems (Bio-Rad).

### Chromatin-immunoprecipitation (CHIP)

SimpleCHIP^®^ Enzymatic Chromatin IP (Cell Signaling) was performed according to manufacturer’s protocol. In brief, IAV-infected Calu-3 cells were fixed with formaldehyde to cross-link proteins to DNA. Next, chromatin was digested with Micrococcal Nuclease into 150–900 bp DNA/protein fragments. Nuclear membrane was broken by sonication. A portion of chromatin preparation was purified for DNA prior to immunoprecipitation to confirm the digestion by electrophoresis and concentration by OD260. One μg rabbit anti-mouse Plscr1 polyclonal antibody, negative control normal rabbit IgG, or positive control Histone H3 rabbit monoclonal antibody was added to 5μg chromatin preparation. The complex co-precipitated and was captured by Protein G magnetic beads. After 4 low salt washes and 2 high salt washes, chromatin was eluted and cross-links were reversed with proteinase K. DNA was purified using spin columns and analyzed by both standard PCR and qRT-PCR. For qRT-PCR, percent input method was used according to the following formula: Percent Input = 2% × 2^(C[T] 2%Input Sample – C[T] IP Sample)^.

### Transformation of competent cells

All *PLSCR1* plasmids were provided by Dr. John MacMicking’s Lab at Yale University. Plasmid DNA concentrations were determined using a spectrophotometer. Fifty ul of DH5α competent cells (Invitrogen) and 50ng of each plasmid was mixed and incubated for 30 minutes on ice. Then, the plasmid and competent cell mixtures were heat shocked for 45 seconds at 42°C, and cooled on ice for 2 minutes. Super Optimal broth with Catabolite repression (SOC) media (homemade with 2% tryptone, 0.5% yeast extract, 10mM NaCl, 2.5mM KCl, 10 mM MgCl2, 10 mM MgSO4, and 20 mM glucose) was added to bacteria. The cells were grown in a shacking incubator at 200RPM for 45 minutes at 37°C. The mixtures were then plated on LB agar ampicillin plates and incubated at 37°C overnight.

Single colonies were selected for each plate, inoculated in Super Optimal Broth (SOB) media (homemade with 2% tryptone, 0.5% yeast extract, 10mM NaCl, 2.5mM KCl, 10 mM MgCl2, and 10 mM MgSO4) and grown to OD600 of 0.6 at 37°C for 12–18 hours, with vigorous shacking of 200RPM. Cultures were then centrifuged at 6000G for 15 minutes at 4°C. Cell pallets were used for plasmid isolation.

### DNA plasmid isolation

DNA plasmids were isolated using the QIAfilter Maxi Cartridges and QIAGEN Plasmid Maxi Kit, according to the manufacture’s protocol. In brief, *PLSCR1*-transduced competent cell pallets were resuspended in buffer P1 containing RNase A solution and lyse blue. After adding Buffer P2, mixtures were incubated at RT for up to 5 minutes. Buffer P3 was then mixed with the lysates, transferred into the barrel of the QIAfilter cartridges and incubated at RT for 10 minutes. Cell lysates were filtered through the equilibrated QIAGEN-tip by gravity. DNA was eluted with Buffer QF and precipitated with isopropanol by centrifuging at 15000G for 10 minutes. Pallets were airdried for 10 minutes and dissolved in di-water. A spectrophotometer was used to determine plasmid concentrations.

### Lentiviral packaging

293T cells were grown in DMEM until 90–95% confluency and then switched to Opti-MEM for 2 hours. Ten ug of plasmid of interest was mixed with lipofectamine 300 transfection reagent (Invitrogen), pPACKH1 HIV Lentivector Packaging Kit (System Biosciences) and Opti-MEM, and incubated for 20mins at RT. After removing half media from the cell culture dishes, DNA-lipid complex was added and cells were incubated at 37°C with 5% CO2 for 6 hours. After 6 hours, culture supernatants were replaced with viral harvesting media (DMEM with glutamine, 10% FBS, 1% MEM non-essential amino acids, 1mM sodium pyruvate, 2% BSA, and 1:50 Hank’s solution) for overnight incubation. Viral supernatant was collected every day for the next 4 days by centrifuging cell culture supernatant at 1200G for 5 minutes. Viral supernatant was mixed with lentiviral concentrator solution (40% polyethylene glycol 8000 and 1.2M NaCl in PBS) at 4:1, and centrifuged at 1600G for 60 minutes at 4°C. The viral pellets were resuspended with Opti-MEM and stored at -80°C.

### Lentiviral transduction

*PLSCR1*^−/−^ A549 cells were seeded in a 96 well plate with DMEM. Lentiviral stocks were serially diluted in DMEM and polybrene (Millipore Sigma) mixture from 1:10 to 1:10,000, and added to cell culture. The 96 well plate was centrifuged at 2000RPM for 30 minutes at RT and incubated at 37°C with 5% CO_2_ overnight. Media was replaced with fresh DMEM without polybrene the next day. After 48 hours post transduction, DMEM with 200ug/ml hygromycin was added for antibiotic selection for 10 days.

### Flow cytometry

Antibodies were titrated prior to confirming transduction efficiency by flow cytometry. One million cells were used for each sample. Samples were stained with LIVE/DEAD™ fixable violet dead cell stain for 405 nm excitation (1:1000, Invitrogen) for 30 minutes. Surface and cytoplasm staining samples were fixed with 4% paraformaldehyde for 20 minutes at RT. Nuclear staining samples were fixed with true nuclear fix concentrate (BioLegend) for 45 minutes at RT. All samples were stained with mouse anti-human PLSCR1 monoclonal antibody (1:500) for 20 minutes, and chicken anti-mouse Alexa 594 (1:1000) for 20 minutes on ice. After fixation and each antibody incubation, surface staining samples were washed twice with FACS buffer (1% bovine serum albumin), cytoplasm staining samples were washed twice with Perm/Wash buffer (BD), and nuclear staining samples were washed twice with true nuclear perm buffer (BioLegend). Final samples were resuspended in FACS buffer. Flow cytometry analysis was performed with FACSAriaIIIu (BD) by Brown University Flow Cytometry and Sorting Facility.

### Cell coverage assay

After IAV infection, A549 cells in 12-well plates were fixed with 4% paraformaldehyde in PBS for 1 hour at RT. Cells were then stained with 1% crystal violet solution (Sigma-Aldrich) for 5 minutes, washed with di-water 3 times and air-dried. Plates were scanned with an APEXVIEW APX100 microscope. Brightfield images were converted to 8-bit and the threshold was adjusted using a dark background in ImageJ[48]. Areas of interest were selected using oval selection. Cell coverages were quantified by measuring mean grey values of each well.

### Bulk RNA sequencing

Selected RNA isolated from mouse lungs in the same treatment group were pooled together into one sample. Pooled RNA concentrations were measured using a Nanodrop (Thermo Scientific). Quality control, library preparation, bulk RNA sequencing and data analysis was performed by Azenta Genomics/GENEWIZ. Heatmaps were made with expressions of selected genes in Morpheus (https://software.broadinstitute.org/morpheus).

### Single cell RNA sequencing analysis

Single cell RNA sequencing dataset were generated from uninfected or IAV-infected mouse lungs at 0, 1, 3, 6 and 21 dpi as previously described[25]. The 10X gene-expression data were processed and normalized using CellRanger (v.3.0.2, 10X Genomics)[25]. Scaling, principal component analysis, dimensionality reduction and cell clustering were performed using Seurat algorithm as previously described[25, 49]. Cell clusters were annotated using known markers from the literature (Table S2).

All following analysis were performed using R Statistical Software (v4.3.0)[50]. Two-dimensional UMAPs were generated with DimPlot (Seurat)[49]. Violin plots of *Plscr1* expression in different cell clusters were plotted with VlnPlot (Seurat)[49]. Differentially expressed genes for ciliated epithelial cell clusters at different timepoints in comparison to 0 dpi were identified with FindMarkers (Seurat)[49]. Those genes were used for Gene Ontology (GO) analysis with enrichGo (clusterProfiler)[51], and heatmaps with DoHeatmap (Seurat)[49].

### Quantitation and statistical analysis

Data were analyzed on GraphPad Prism software. Kaplan-Meier survival analysis was used to compare survival rates. Ordinary two-way ANOVA tests were used to compare weight losses. Statistical significance of differences was assessed using the parametric Student’s two-tailed t tests for all other normally distributed data. Differences were considered significant when p < 0.05. Outlier tests with the ROUT method (Q = 1%) were used to identify and remove any outliers.

## Supplementary Material

1
