# Supplementary material for: Phospholipid Scramblase 1 (PLSCR1) Regulates Interferon-Lambda Receptor 1 (IFN-λR1) and IFN-λ Signaling in Influenza A Virus (IAV) Infection
Source: bioRxiv. 2024 Nov 21:2024.11.20.624469. Preprint. [Version 1] doi: 10.1101/2024.11.20.624469 (PMC11601549; doi:10.1101/2024.11.20.624469)

bioRxiv preprint doi: https://doi.org/10.1101/2024.11.20.624469; this version posted November 21, 2024. The copyright holder for this preprint (which was not certified by peer review) is the author/funder, who has granted bioRxiv a license to display the preprint in perpetuity. It is made available under aCC-BY 4.0 International license.

qRT-PCR primers		
**Gene**	**Primer**	**Sequence (5'-3')**
IAV M gene	Fwd	CATGGAATGGCTAAAGACAAGACC

bioRxiv preprint doi: https://doi.org/10.1101/2024.11.20.624469; this version posted November 21, 2024. The copyright holder for this preprint (which was not certified by peer review) is the author/funder, who has granted bioRxiv a license to display the preprint in perpetuity. It is made available under aCC-BY 4.0 International license.

	Rev	CCATTAAGGGCATTTTGGACA
Plscr1	Fwd	GCCCAAGTTCACTCTCCAAA
	Rev	GAGCTCAAAGTCAATGTCGG
Ifn-α	Fwd	AGTGAGCTGACCCAGCAGAT
	Rev	AGACAGCCTTGCAGGTCATT
Ifn-β	Fwd	CCCTATGGAGATGACGGAGA
	Rev	ACCCAGTGCTGGAGAAATTG
Ifn-γ	Fwd	ATGGCTATTTCTGGCTGTTACT
	Rev	AATGACGCTTATGTTGTTGCTG
Ifn-λ2 and *Ifn-λ3*	Fwd	AGTGGAAGCAAAGGATTG
	Rev	GAGATGAGGTGGGAACTG
Ifn-λr1	Fwd	GACGAGTACAGGCAGCTTCC
	Rev	AGCATTGACCCTTAGGATCTTCTC
Gapdh	Fwd	AGGTCGGTGTGAACGGATTTG
	Rev	TGTAGACCATGTAGTTGAGGTCA
PLSCR1	Fwd	CTGACTTCTGAGAAGGTTGC
	Rev	GAATGCTGTCGGTGGATACTG
IFN-λ1	Fwd	ACATTGGCAGGTTCAAATCTC
	Rev	TGAGTGACTCTTCCAAGGC
IFN-λR1	Fwd	CAGTGTCCCGAAATACAGCAAG
	Rev	TGTGTCCAGAAAAGTCCAGGGC
IFN-λR1 promoter	Fwd	CCGGCCTTGAACTCTCCCT
	Rev	GCGCTCGAAACTCGCCC
GAPDH	Fwd	TCGTGGAAGGACTCATGACC
	Rev	TCCACCACCCTGTTGCTGTA

Table S1. PCR primer list.

bioRxiv preprint doi: https://doi.org/10.1101/2024.11.20.624469; this version posted November 21, 2024. The copyright holder for this preprint (which was not certified by peer review) is the author/funder, who has granted bioRxiv a license to display the preprint in perpetuity. It is made available under aCC-BY 4.0 International license.

scRNA-seq cluster annotations

**Cluster**	**Cell Type**	**Transcriptional Marker**
0	AT2 Cells	*Epcam, Sftpc*
1	Mfap5+ Fibroblasts	*Pdgfra, Mfap5*
2	Microvascular Endothelial Cells	*Pecam1, Gpihbp1*
3	Damage-Responsive Fibroblasts	*Pdgfra, Wt1*
4	T Cells	*Ptprc, Cd3e*
5	Activated Microvascular Endothelial Cells	*Pecam1, Gpihbp1, S1pr1*

bioRxiv preprint doi: https://doi.org/10.1101/2024.11.20.624469; this version posted November 21, 2024. The copyright holder for this preprint (which was not certified by peer review) is the author/funder, who has granted bioRxiv a license to display the preprint in perpetuity. It is made available under aCC-BY 4.0 International license.

6	Airway Smooth Muscle Cells	*Hhip, Acta2*
7	IAV-Infected Epithelial Cells	*Epcam, Flu*
8	Microvascular Endothelial Cells	*Pecam1, Gpihbp1*
9	High Mitochondrial Content Cells	
10	Krt8+ Epithelial Cells	*Epcam, Krt8*
11	AT2 Cells	*Epcam, Sftpc*
12	Ciliated Epithelial Cells	*Epcam, Foxj*
13	Club Cells	*Epcam, Scgb3a2*
14	B Cells	*Ptprc, Cd19*
15	Myofibroblasts	*Pdgfra, Acta2*
16	Neutrophils	*Ptprc, Mmp9*
17	Alveolar Fibroblasts	*Pdgfra, Wnt2*
18	Epcam+Pecam1+ Cells	*Epcam, Pecam1*
19	Monocytes	*Ptprc, Ly6c2*
20	Interferon-Responsive Fibroblasts	*Pdgfra, Bst2*
21	Mesothelial Cells	*Msln, Wt1*
22	Epcam+Col1a2+ Cells	*Epcam, Col1a2*
23	Interstitial Macrophages	*Ptprc, Cd68, C1qb*
24	Lymphatic Endothelial Cells	*Pecam1, Prox1*
25	Aerocytes	*Pecam 1, Car4*
26	Vascular Smooth Muscle Cells	*Acta2, Pdgfrb, Notch3*
27	AT1 Cells	*Epcam, Akap5, Ager*
28	Macrovascular Endothelial Cells	*Pecam1, Vwf*
29	Ciliated Epithelial Cells	*Epcam, Foxj1*

bioRxiv preprint doi: https://doi.org/10.1101/2024.11.20.624469; this version posted November 21, 2024. The copyright holder for this preprint (which was not certified by peer review) is the author/funder, who has granted bioRxiv a license to display the preprint in perpetuity. It is made available under aCC-BY 4.0 International license.

30	Alveolar Macrophages	*Ptprc, Cd68, Chil3*
31	NK Cells	*Ptprc, Klrb1c*
32	Epcam+Col1a2+ Cells	*Epcam, Col1a2*
33	Immune Doublets	*Ptprc, Cd3e, Cd19*
34	Regulatory T cells	*Cd4, Il2ra*
35	Basophils/Mast Cells	*Ptprc, Fcer1a, Gata2*
36	Multiplets	
37	Platelets	

Table S2. ScRNA-seq Cluster Annotations.

(which was not certified by peer review) is the author/funder, who has granted bioRxiv a license to display the preprint in perpetuity. It is m
available under aCC-BY 4.0 International license.

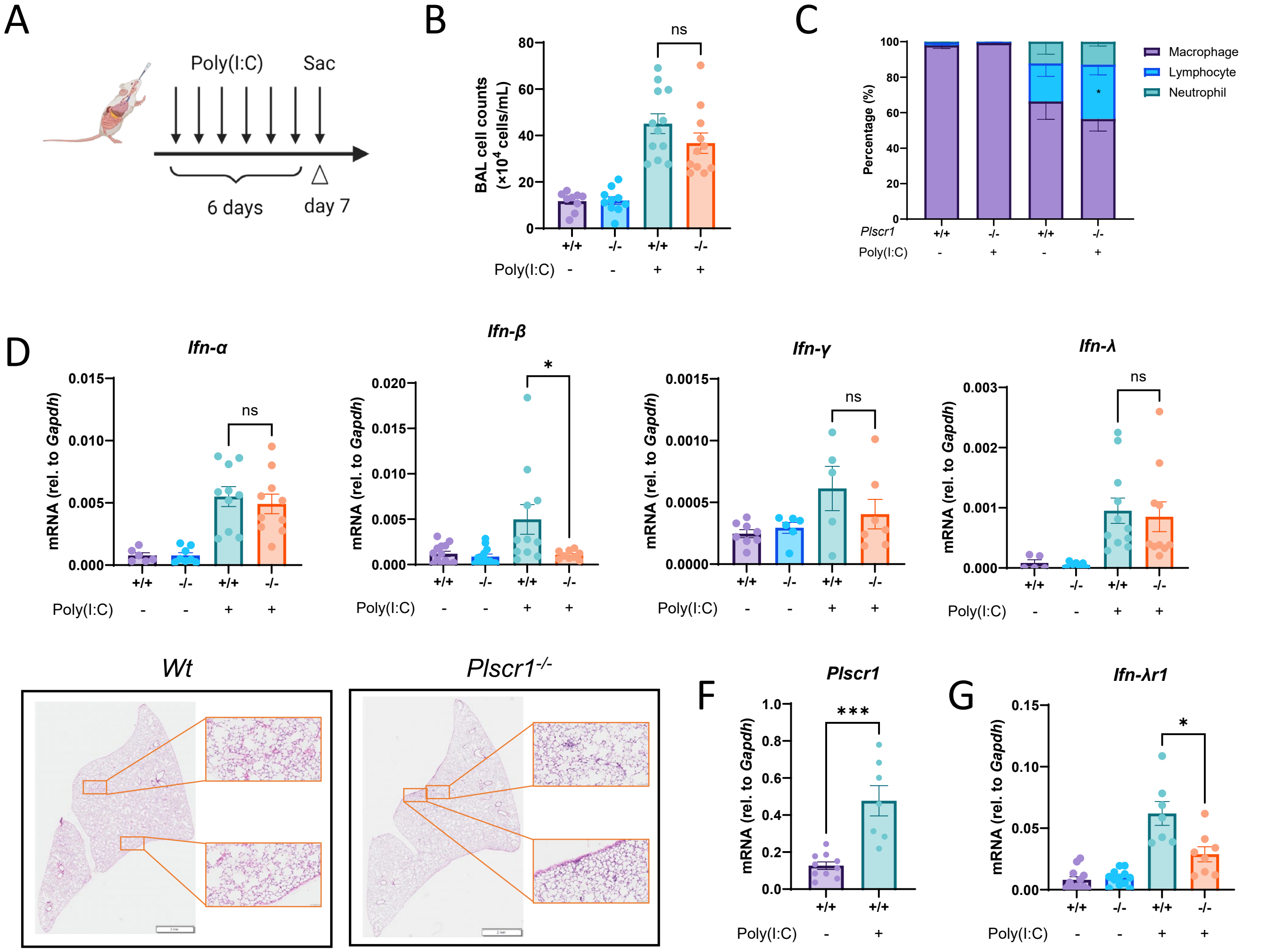

Supplemental Figure 1. Requirement of *Plscr1* in IFN-λ Signaling Independent of Viral Titer
Wt and *Plscr1*$^{-/-}$ mice were intranasally given 2.5 µg/g of body weight of poly(I:C) (HMW) constitutively for 6 days and sacrificed on day 7.
(A) Scheme of experiment.
(B) Total BAL leukocyte numbers.
(C) Differential cell counts in BAL.
(D, F-G) Whole lungs were analyzed for *Ifn-α*, *Ifn-β*, *Ifn-γ*, *Ifn-λ* (D); *Plscr1* (F); and *Ifn-λr1* (G) RNA by qRT-PCR.
(E) Representative lung sections stained with H&E. Scale bars represent 3 mm (main) and 200 µm (inlays).
Data are expressed as mean ± SEM of n = 5-12 mice/group. All data were pooled from three independent experiments. ns, not significant, *p < 0.05, ***p<0.001.

(which was not certified by peer review) is the author/funder, who has granted bioRxiv a license to display the preprint in perpetuity. It is m
available under aCC-BY 4.0 International license.

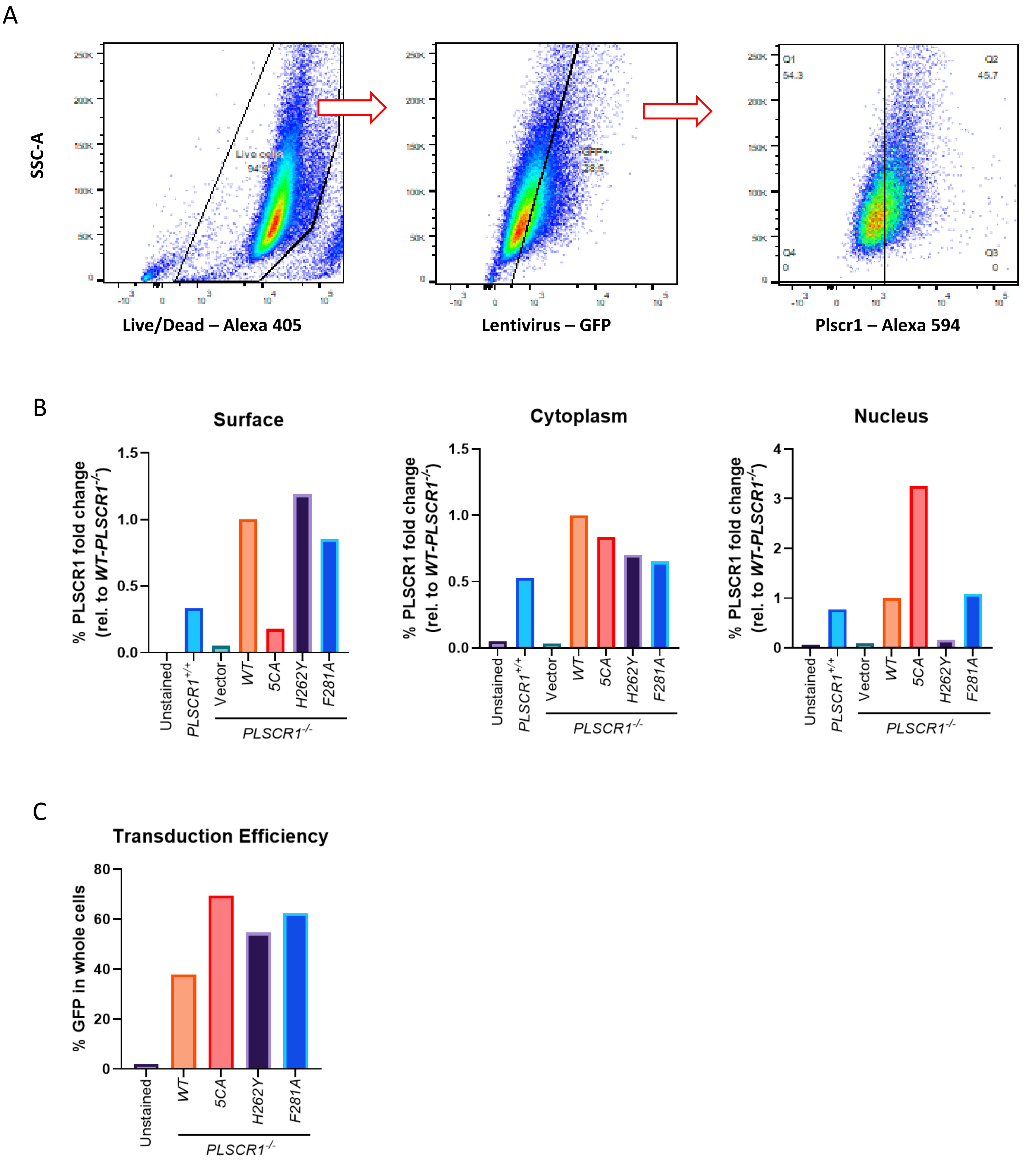

Supplemental Figure 2. PLSCR1 Transduction Efficiency and Distribution

PLSCR1 plasmids on PLV-EF1a-IRES-Hygro backbone were packaged into GFP-expressing lentivirus. *PLSCR1*$^{-/-}$ A549 cells were transduced using lentivirus. After a 10-day hygromycin selection, cells were analyzed using flow cytometry.

(A) Gating strategy for live, GFP+ and PLSCR1+ A549 cells.

(B) Surface, cytoplasm and nuclear expression of PLSCR1. Data are presented as fold change compared to *WT-PLSCR1*$^{-/-}$ A549 cells.

(C) Lentiviral transduction efficiency.

(which was not certified by peer review) is the author/funder, who has granted bioRxiv a license to display the preprint in perpetuity. It is m
available under aCC-BY 4.0 International license.

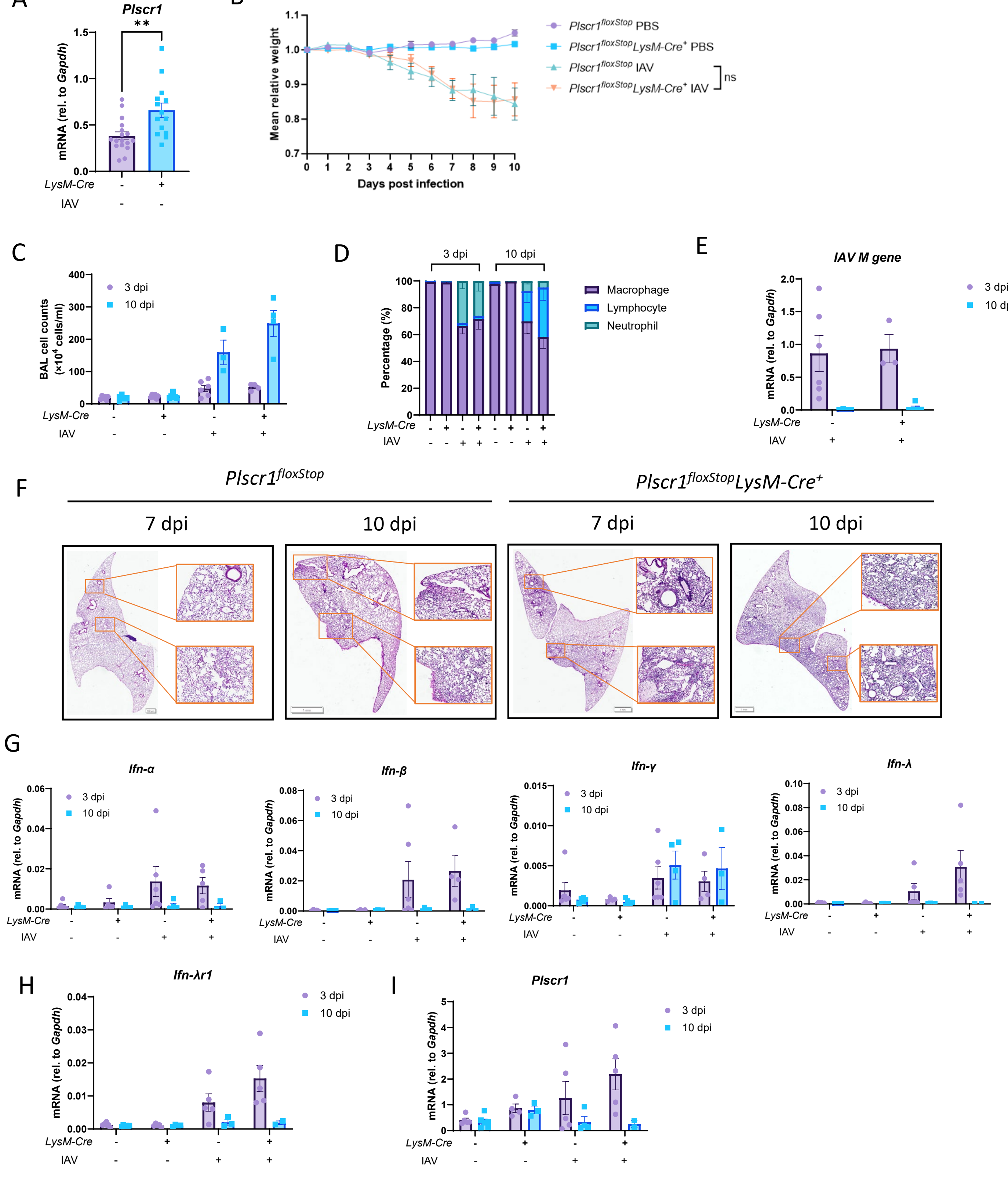

Supplemental Figure 3. Unaffected Susceptibility of $Plscr1^{floxStop}LysM\text{-}Cre^{+}$ Mice to Influenza Virus Infection

$Plscr1^{floxStop}$ and $Plscr1^{floxStop}LysM\text{-}Cre^{+}$ mice were exposed to sublethal (300 pfu) IAV (WSN) infection.

(A) Validation of *Plscr1* overexpression in lungs of $Plscr1^{floxStop}LysM\text{-}Cre^{+}$ mice by qRT-PCR.

(B) Mean relative weight of mice.

(C) Total BAL leukocyte numbers.

(D) Differential cell counts in BAL.

(E) Viral RNA load in the lungs was assessed by quantifying M gene by qRT-PCR.

(F) Representative lung sections stained with H&E.

(G-I) Whole lungs were analyzed for *Ifn-α*, *Ifn-β*, *Ifn-γ*, *Ifn-λ* (G); *Ifn-λr1* (H); and *Plscr1* (I) RNA by qRT-PCR.

Data are expressed as mean ± SEM of n = 15-16 mice/group for weight loss. For the rest analysis, n= 3-7 mice/group. All data were pooled from three independent experiments. ns, not significant, $*p < 0.05$, $**p < 0.01$. dpi, days post infection.

(which was not certified by peer review) is the author/funder, who has granted bioRxiv a license to display the preprint in perpetuity. It is m
available under aCC-BY 4.0 International license.

Source Fig. 3G

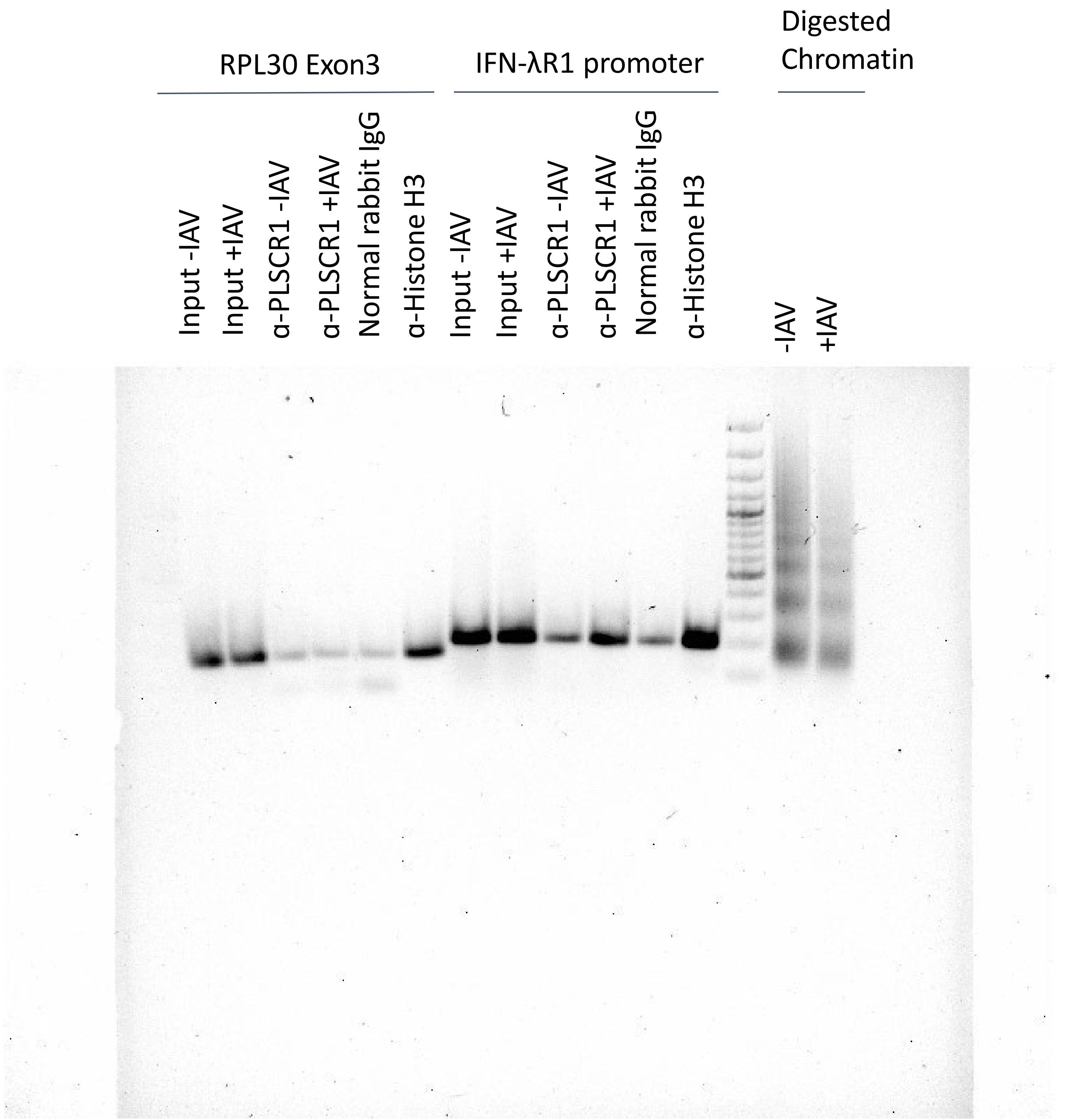

(which was not certified by peer review) is the author/funder, who has granted bioRxiv a license to display the preprint in perpetuity. It is m
available under aCC-BY 4.0 International license.

Source Fig. 4A

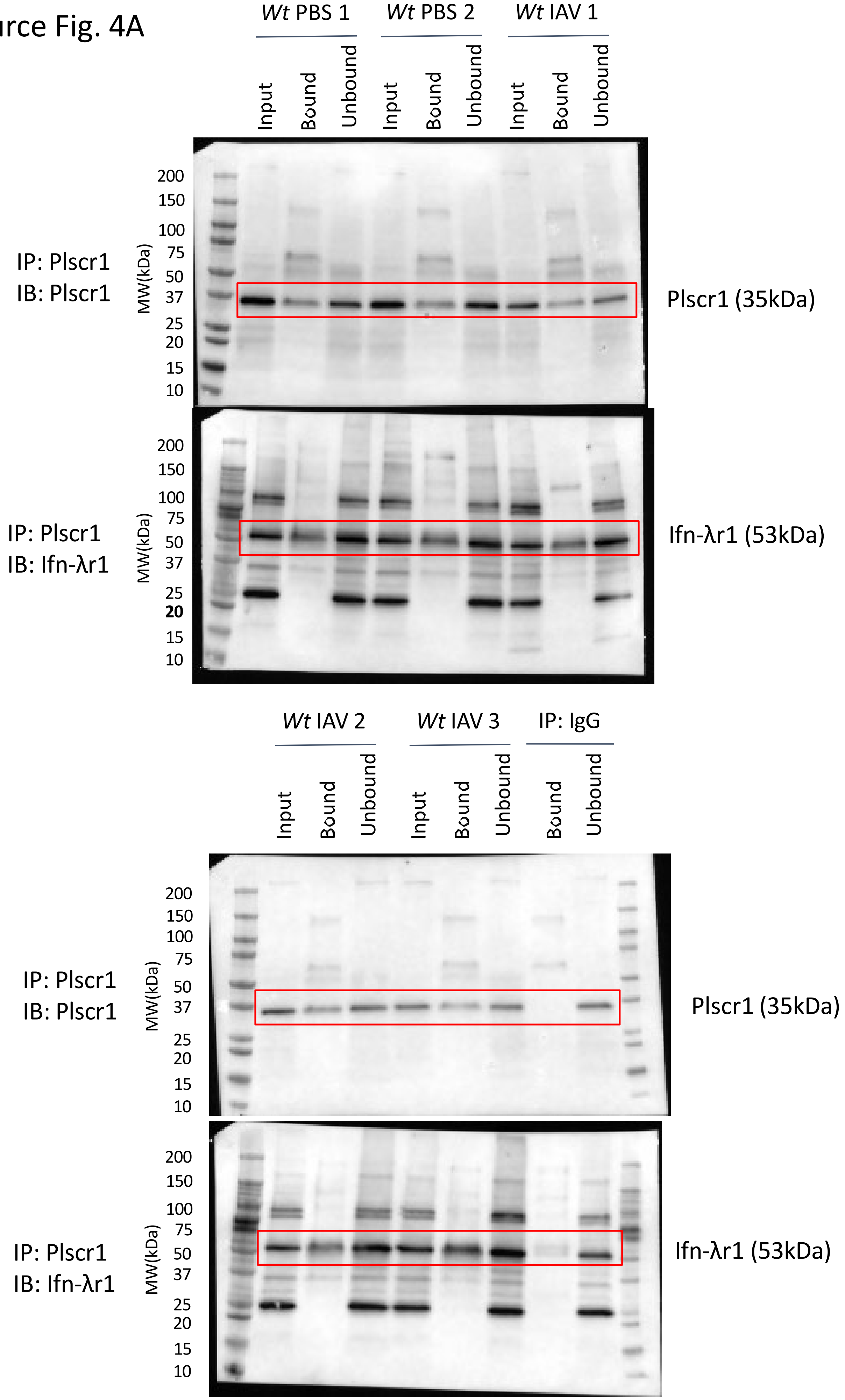

Supplement: 1 [file NIHPP2024.11.20.624469v1-supplement-1.pdf]
